# Air Pollution, Socioeconomic Status, and Age-Specific Mortality Risk in the United States

**DOI:** 10.1001/jamanetworkopen.2022.13540

**Published:** 2022-05-24

**Authors:** Antonio Fernando Boing, Priyanka deSouza, Alexandra Crispim Boing, Rockli Kim, S. V. Subramanian

**Affiliations:** 1 Post-Graduate Program in Public Health, Federal University of Santa Catarina, Florianópolis, Brazil; 2 Urban and Regional Planning Department, University of Colorado Denver, Denver; 3 Division of Health Policy and Management, College of Health Sciences, Korea University, Seoul, South Korea; 4 Interdisciplinary Program in Precision Public Health, Department of Public Health Sciences, Graduate School of Korea University, Seoul, South Korea; 5 Harvard Center for Population and Development Studies, Cambridge, Massachusetts; 6 Department of Social and Behavioral Sciences, Harvard T.H. Chan School of Public Health, Boston, Massachusetts

## Abstract

**Importance:**

Prior studies on the association between fine particulate matter with diameters 2.5 μm or smaller (PM_2.5_) and probability of death have not applied multilevel analysis disaggregating data for US census tract, states, and counties, nor tested its interaction by socioeconomic status (SES). Such an approach could provide a more refined identification and targeting of populations exposed to increased risk from PM_2.5_.

**Objective:**

To assess the association between PM_2.5_ and age-specific mortality risk (ASMR) using disaggregated data at the census tract level and evaluate such association according to census tract SES.

**Design, Setting, and Participants:**

This nationwide cross-sectional study used a linkage of 3 different data sets. ASMR for the period of 2010 to 2015 was obtained from the National Center for Health Statistic, SES data covering a period from 2006 to 2016 came from the American Community Survey, and mean PM_2.5_ exposure levels from 2010 to 2015 were derived from well-validated atmospheric chemistry and machine learning models. Data were analyzed in April 2021.

**Exposures:**

The main exploratory variable was mean census tract–level long-term exposure to PM_2.5_ from 2010 to 2015.

**Main Outcomes and Measures:**

The primary outcome was census tract–level ASMR. Multilevel models were used to quantify the geographic variation in ASMR at levels of census tract, county, and state. Additional analysis explored the interaction of SES in the association of ASMR with PM_2.5_ exposure.

**Results:**

Data from 67 148 census tracts nested in 3087 counties and 50 states were analyzed. The association between exposure to PM_2.5_ and ASMR varied substantially across census tracts. The magnitude of such association also varied across age groups, being higher among adults and older adults. Census tracts accounted for most of the total geographic variation in mortality risk (range, 77.0%-94.2%). ASMR was higher in deciles with greater PM_2.5_ concentration. For example, ASMR for age 75 to 84 years was 54.6 per 1000 population higher in the decile with the second-highest PM_2.5_ concentration than in the decile with the lowest PM_2.5_ concentration. The ASMR, PM_2.5_ concentrations, and magnitude of the association between both were higher in the census tracts with the lowest SES.

**Conclusions and Relevance:**

This cross-sectional study found that census tracts with lower SES presented higher PM_2.5_ concentrations. ASMR and air pollution varied substantially across census tracts. There was an association between air pollution and ASMR across all age groups in the United States. These findings suggest that equitable public policies aimed at improving air quality are needed and important to increase life expectancy.

## Key Points

**Question:**

What are the associations among exposure to fine particulate matter with diameters 2.5 μm or smaller (PM_2.5_), age-specific mortality risk (ASMR), and socioeconomic status (SES) when disaggregating data for US census tracts, states and counties?

**Findings:**

This cross-sectional study that included data from 67 148 census tracts found associations between PM_2.5_ and ASMR across all age groups and that the magnitude of this association was higher in the census tracts with lowest SES.

**Meaning:**

These findings suggest that air pollution is an important factor associated with mortality, and public policies aiming to reduce mortality and inequalities must take small geographic units, like census tracts, into account.

## Teaser

This cross-sectional study assesses associations among fine particulate matter 2.5 μm or smaller, age-specific mortality risk, and socioeconomic status.

## Introduction

Exposure to ambient fine particulate matter (mass concentrations of particles ≤2.5 μm in diameter [PM_2.5_]) is associated with increased mortality and morbidity and reduced life expectancy.^1,2,3,4^ Key findings on the negative outcomes associated with long-term exposure to PM_2.5_ have come from cohort studies that have followed fixed sets of individuals over time.^5,6,7^ In most studies, these individuals are not representative of the national US population. Studies relying on administrative data that are more representative of the US population as a whole have also found overwhelming evidence of negative outcomes associated with long-term exposure to PM_2.5_^8,9,10,11^ and the public health benefits associated with the overall decline of PM_2.5_ concentrations over the years.^12,13,14^ Although ecological regression analyses relying on administrative data have several limitations, eg, they are unable to adjust for individual-level risk factors and confusion between ecological associations and individual-level associations may present an ecological fallacy, they still may allow us to draw conclusions about the outcomes associated with pollution at the area level, which are important for policy makers.^15^

It is also well known that exposure to particulate matter pollution varies substantially by geography and socioeconomic status (SES).^16,17^ Economic activity, topography, climatic conditions, and industrial and population density in a region are important factors that modulate PM_2.5_ emissions.^14^ Environmental justice advocates have long pointed out that in the US, Black individuals experience disproportionately higher PM_2.5_ concentrations than White individuals.^18^ A 2020 study by Colmer et al^17^ analyzed air pollution in 1981 and 2016 across US census tracts and reported that although absolute disparities have decreased, relative disparities in exposure continue to persist.

Mortality levels in different age groups also significantly vary over geography and SES in the US. Few studies have investigated the magnitude of mortality and its association with air pollution by disaggregating data to smaller geographic spaces, such as census tracts, despite research showing that the finer the spatial resolution of the study, the more pronounced were disparities in PM_2.5_ exposure by race and ethnicity.^19^ One such study found that in the state of New Jersey, the association of PM_2.5_ with mortality was significantly modified in census tracts with more Black residents, lower home values, and lower median incomes.^10^ However, these studies failed to explicitly model the multilevel structure of the data. Recent studies have demonstrated that the total variance of life expectancy in the US is distributed very differently across geographical regions, and not incorporating such data hierarchy in an analysis limits the interpretability of study findings.^20,21^ To our knowledge, there have been no previous studies that have analyzed the probability of death and its association with PM_2.5_ via disaggregating data for census tracts and applying multilevel analysis.

This study aims to partition the variations in age-specific mortality risk (ASMR) and PM_2.5_ at 3 geographic scales: states, counties, and census tracts. We also explore the interaction of SES in the association between PM_2.5_ and ASMR using multilevel models. Our approach thus enables a more precise identification of populations with higher risk in terms of geographic scale and SES.

## Methods

The Harvard Longwood Campus Institutional Review Board (IRB) allows researchers to self-determine when an IRB application is required using an IRB Decision Tool. This project used publicly accessible secondary data obtained from the US Small-Area Life Expectancy Estimates Project^22^ and the Opportunity Insights^24^ database. These activities did not meet the regulatory definition of human participants research. As such, an IRB review was not required. This study followed the Strengthening the Reporting of Observational Studies in Epidemiology (STROBE) reporting guideline.

### Age-Specific Mortality Risk

Data for the probability of death in each census tract between ages younger than 1 year, 1 to 4 years, 5 to 14 years, 15 to 24 years, 25 to 34 years, 35 to 44 years, 45 to 54 years, 55 to 64 years, 65 to 74 years, and 75 to 84 years were obtained from the abridged period life tables calculated by the US Small-Area Life Expectancy Estimates Project.^22^ This was a project conducted by the National Center for Health Statistics to estimate census tract–level life expectancy in the US.

Briefly, the population data used to estimate the probability of death were derived from the 2010 decennial census and from the American Community Survey (ACS) for the period 2011 to 2015. The death records that occurred in this period were obtained from the National Vital Statistics System and were geocoded using the residential addresses of decedents to identify the census tracts. Life tables were calculated for each census tract with a minimum pooled population size of 5000 inhabitants in the period (2010-2015). Death counts were available for all age groups (as observed or estimated), with coherent age patterns and SEs of mortality. When zero death counts were observed in any age group in a census tract, the value was replaced with an estimated number based on a combination of demographic, socioeconomic, and geographic characteristics included in the models.^22^ The definition of the probability of death between 2 exact ages is presented in eFigure 1 in the Supplement. A detailed description of the techniques used to calculate the abridged period life tables has been published elswhere.^22^

### Assessment of PM_2.5_ Exposure

PM_2.5_ exposure levels derived from a well-validated atmospheric chemistry and machine learning model^23^ were available at an annual-level on a 0.01° × 0.01° grid resolution across the entire continental US. The model performs well, with a cross-validated *R*^2^ of 0.76. We estimated the census tract–level long-term exposure to PM_2.5_ (mean for the 2010 to 2015 period) by using a spatially weighted mean of the grid cells grid points within a census tract using the exact_extract package in R statistical software version 4.1.2 (R Project for Statistical Computing). We used mean values of PM_2.5_ (2010-2015) at the census tract level as our main exposure by calculating mean estimated PM_2.5_ concentrations within a given tract.

### Socioeconomic and Demographic Covariates

Data on census tract–level SES and demographic factors are from the Opportunity Insights database.^24^ We used 5 socioeconomic and demographic variables at the census tract level: (1) median household income, obtained from the 2012 to 2016 ACS; (2) share of individuals in the tract below the federal poverty line, estimated by the 2006 to 2010 ACS; (3) population density, defined as the number of residents per square mile based on 2010 census data; (4) proportion of Black residents in the census tract, based on the 2010 census; and (5) proportion of people aged 25 years or older who have an undergraduate degree, master’s degree, professional school degree, or doctorate degree, based on 2006 to 2010 ACS data. Different year ranges were used based on the availability of the data.

### Statistical Analysis

All analyses conducted in this study considered the multilevel structure of the data. We analyzed data from 67 148 census tracts at level 1, nested within 3087 counties at level 2, and nested within 50 states at level 3 (eFigure 2 in the Supplement). We present descriptive statistics of the mean and SE of ASMR, as well as the distribution of SES characteristics for each decile of the distribution of mean PM_2.5_ concentration. We also mapped the geographical distribution of all variables included in the study.

ASMR was analyzed as a continuous variable with multilevel linear models, including random effects for states, counties, and census tracts. First, we used null models to estimate the crude variation in ASMR at each level. The proportion of variance attributed to each level was computed as the division of the observed variance at that level by the sum of the observed variances in the 3 analyzed levels. The quotient obtained was multiplied by 100. Then, we included census tract PM_2.5_ and SES and demographic characteristics in models to estimate how much of the variation observed in each level may be explained by these variables. As sensitivity analyses, we also calculated mean PM_2.5_ concentrations from 2000 to 2015, and in addition to exploring PM_2.5_ concentrations in deciles, we ran linear multilevel models using this as a continuous variable. We stratified this analysis for each SES variable for people older than 45 years.

Finally, we mapped the geographical distribution of all analyzed variables. Data were analyzed using Stata version 15.1 (StataCorp), and maps were plotted using QGIS version 3.10.1.0 (QGIS Project). A 2-tailed *P* < .05 was considered significant. Data were analyzed in 2021.

## Results

### Regional Distribution of ASMR, PM_2.5_, and SES Characteristics

Data from 67 148 census tracts nested in 3087 counties and 50 states were analyzed. ASMR varied substantially across census tracts. Large differences were observed in all age groups, from children to older adults. Analyzing the extreme age groups, among children younger than 1 year, the probability ranged from 0.58 deaths per 1000 population (Orange County, California) to 80.90 deaths per 1000 population (Pensacola, Florida). For populations aged between 75 and 84 years, the absolute difference in the probability of death between the 5th percentile and the 95th percentile was 352.56 deaths per 1000 population. The geographic distributions of the probability of death across all age groups are shown in eFigures 3 through 12 in the Supplement.

Similar magnitudes of regional inequalities were observed when analyzing exposure to PM_2.5_ (Figure). The lowest PM_2.5_ concentration values were observed in the West (except for California) and in parts of the Southwest and Midwest of the US. The Alaska census sectors had the lowest PM_2.5_ concentration values (the lowest in Sitka County, at 1.08 μm), whereas the highest PM_2.5_ values were found in California counties (the highest in Fresno County, at 17.49 μm). The geographic distributions of SED variables are shown in eFigures 13 through 17 in the Supplement. The SES variables varied considerably across census tracts. Census tracts in the South tended to be poorer and had higher proportions of Black residents. Median household income was higher in urban areas.

**Figure.**
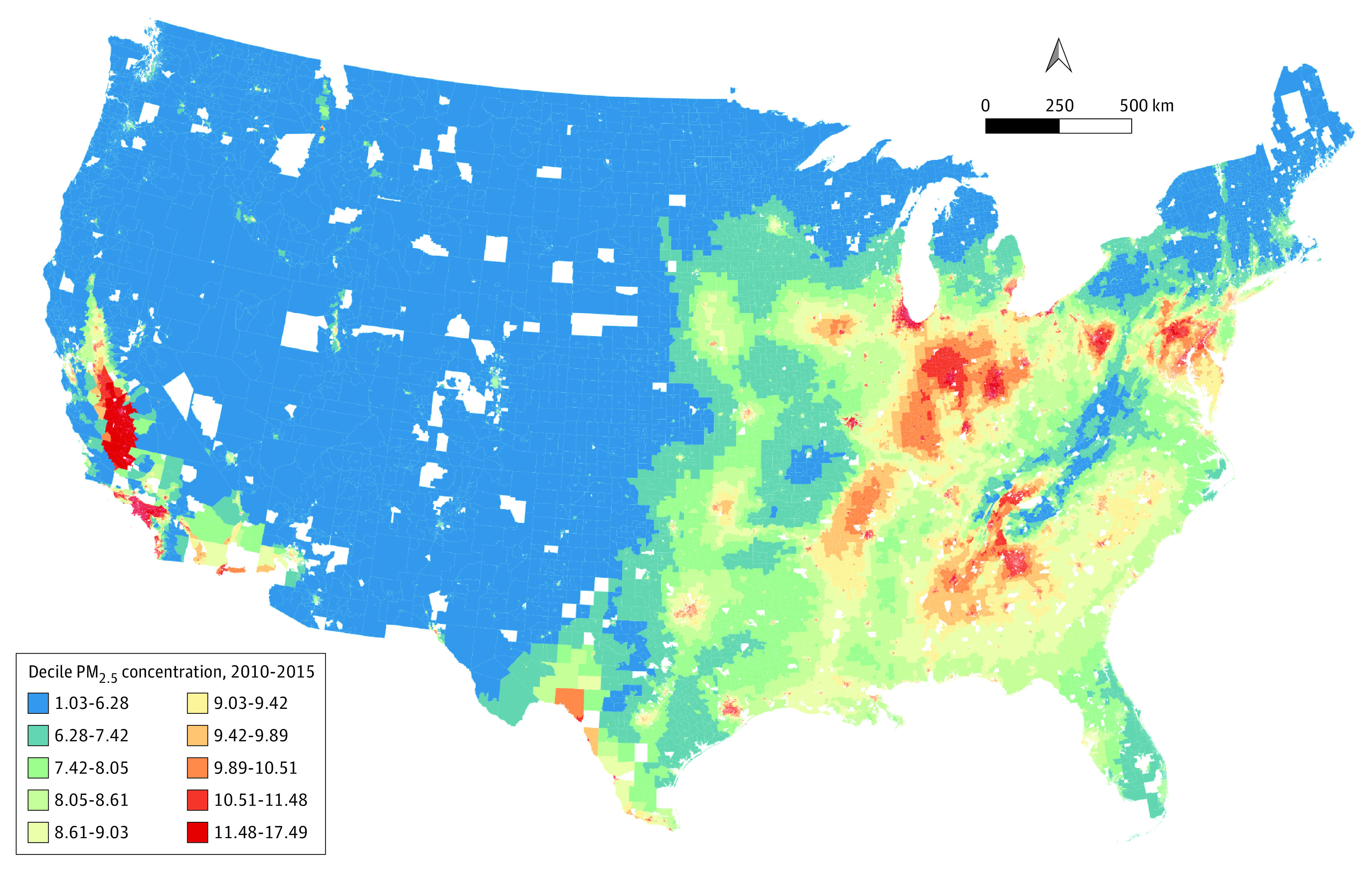
Geographic Distribution of Fine Particulate Matter Concentration (PM_2.5_) in the US by Census Tract, 2010-2015 White areas indicate no available data.

### Partitioning Variation in ASMR

Across all age groups, census tracts accounted for most of the total variation in the ASMR observed (range, 77.02%-94.21%) (Table 1). For children, adolescents, and older adults (>75 years), census tracts accounted for more than 90% of the total variation. SES and PM_2.5_ concentrations contributed an important proportion of the variance observed in the multilevel models, particularly at the state and county scales (66.08%-90.50%) (eTables 1-3 in the Supplement). The variance in ASMR associated with SES and PM_2.5_ concentrations was lower at the census tract level (<10%) for ages younger than 35 years but larger (20%-50%) for older age groups.

**Table 1.** Variance in Probability of Dying by Age Group; Proportion of Variance Attributable to State, County, and Census Tract; and Constant of the Regression Models^a^

Age, y	Total, variance^b^	Census tract		County		State		Regression parameter, constant (SE)
		Variance (SE)^b^	VPC, %	Variance (SE)^b^	VPC, %	Variance (SE)^b^	VPC, %	
<1	42.86	40.12 (0.22)	93.61	1.29 (0.09)	3.01	1.46 (0.32)	3.38	7.92 (0.18)
1-4	3.26	3.01 (0.02)	92.33	0.15 (0.01)	4.60	0.10 (0.02)	3.07	1.76 (0.05)
5-14	3.97	3.74 (0.02)	94.21	0.13 (0.01)	3.27	0.10 (0.02)	2.52	2.00 (0.05)
15-24	30.61	27.18 (0.15)	88.79	2.11 (0.13)	6.89	1.32 (0.30)	4.31	9.35 (0.17)
25-34	75.16	63.12 (0.35)	83.98	7.53 (0.38)	10.02	4.51 (0.98)	6.00	14.058 (0.31)
35-44	171.47	135.45 (0.75)	78.99	18.98 (0.85)	11.07	17.04 (3.58)	9.94	20.536 (0.60)
45-54	637.24	490.78 (2.72)	77.02	65.00 (2.83)	10.20	81.46 (16.90)	12.78	43.942 (1.30)
55-64	1867.30	1452.40 (8.04)	77.78	156.69 (6.90)	8.39	258.21 (53.56)	13.83	86.150 (2.31)
65-74	4643.80	3785.11 (20.94)	81.51	347.66 (16.24)	7.49	511.03 (106.45)	11.00	173.602 (3.26)
75-84	11 710.34	10 500.97 (57.96)	89.67	446.84 (27.82)	3.82	762.53 (160.14)	6.51	386.622 (4.00)

Abbreviation: VPC, variance partitioning coefficient.

^a^ All estimates from null 3-level model.

^b^ Estimates multiplied by 1000.

### Association Between PM_2.5_ and SES Variables

We observed a profound difference in the racial composition of the census tracts based on which PM_2.5_ decile they were in (Table 2). In the lowest PM_2.5_ decile, only 1.80% of residents were Black, whereas the percentage of Black residents increased to 23.50% and 17.75% in the 2 deciles with the highest concentration of PM_2.5_.

**Table 2.** Socioeconomic and Demographic Variables According to Deciles of Fine Particulate Matter Concentration

Decile	Mean (SE)				
	%		Household income, mean, $	Residents below the federal poverty line. %	Population density, residents per mile^2^
	Black people	People aged 25 or older with college degree			
					
1	1.80 (0.04)	25.96 (<0.01)	59 825.90 (295.11)	12.05 (0.10)	793.11 (18.78)
2	4.08 (0.08)	27.40 (<0.01)	63 601.42 (337.00)	11.32 (0.10)	1700.70 (33.84)
3	9.73 (0.19)	25.77 (<0.01)	58 172.21 (327.70)	13.24 (0.12)	2618.56 (52.34)
4	11.11 (0.20)	25.21 (<0.01)	58 518.01 (342.26)	13.62 (0.12)	2737.88 (67.31)
5	14.66 (0.23)	25.56 (<0.01)	58 720.66 (358.68)	14.36 (0.14)	2965.19 (71.26)
6	18.21 (0.30)	27.20 (<0.01)	61 237.31 (374.60)	14.55 (0.14)	8841.34 (210.06)
7	18.34 (0.29)	28.30 (<0.01)	61 537.39 (408.21)	15.13 (0.15)	9829.97 (247.24)
8	21.99 (0.34)	28.66 (<0.01)	59 733.97 (382.86)	15.24 (0.15)	6957.42 (193.92)
9	23.50 (0.36)	26.15 (<0.01)	54 591.77 (348.92)	17.27 (0.17)	6245.76 (154.95)
10	17.75 (0.34)	24.58 (<0.01)	55 520.32 (351.73)	17.93 (0.16)	9164.28 (106.19)

We also observed an important difference when we analyzed the proportion of residents below the federal poverty line and population density, with higher values observed in the highest air pollution deciles (eTable 4 in the Supplement). The adjusted model demonstrated that higher population density, higher proportions of Black residents and residents below the poverty line, lower household income, and lower education levels were associated with higher PM_2.5_ concentrations.

### Association Between ASMR and PM_2.5_

ASMR in census tracts also varied significantly based on the corresponding PM_2.5_ deciles (Table 3). These probabilities were higher in the highest deciles of air pollution in all age groups. The greatest absolute difference was observed between decile 9 of PM_2.5_ (second highest) and decile 1 of PM_2.5_ (lowest) in the group aged 75 to 84 years, at 54.59 deaths per 1000 population. In relative terms, we highlight that in the group aged 45 to 54 years, the probability of death was 45% higher in decile 9 compared with decile 1. Interestingly, in all ages, the probability of death was greater in decile 9 and decile 8 than decile 10.

**Table 3.** Age-Specific Probability of Dying by Deciles of Fine Particulate Matter 2.5 μm or Smaller Concentration

Decile	ASMR by age group, mean (SE) × 1000									
	≤1 y	1-4 y	5-14 y	15-24 y	25-34 y	35-44 y	45-54 y	55-64 y	65-74 y	75-84 y
										
1	6.91 (0.07)	1.59 (0.02)	1.84 (0.02)	8.98 (0.07)	12.91 (0.10)	17.58 (0.14)	34.34 (0.23)	69.98 (0.37)	148.02 (0.64)	356.81 (1.19)
2	7.00 (0.07)	1.56 (0.02)	1.82 (0.02)	8.36 (0.06)	12.22 (0.06)	16.93 (0.13)	36.48 (0.25)	74.66 (0.41)	156.51 (0.70)	372.48 (1.24)
3	7.60 (0.08)	1.68 (0.02)	1.87 (0.02)	8.77 (0.07)	13.07 (0.07)	18.85 (0.15)	41.81 (0.29)	83.90 (0.46)	168.72 (0.75)	383.29 (1.30)
4	7.92 (0.08)	1.71 (0.02)	1.94 (0.02)	8.77 (0.06)	13.18 (0.06)	19.61 (0.15)	43.51 (0.29)	87.43 (0.48)	176.70 (0.76)	392.28 (1.29)
5	8.16 (0.08)	1.70 (0.02)	1.97 (0.02)	8.86 (0.06)	13.22 (0.06)	19.84 (0.15)	44.44 (0.30)	89.45 (0.49)	179.60 (0.80)	396.50 (1.30)
6	7.77 (0.08)	1.57 (0.02)	1.77 (0.02)	8.26 (0.06)	12.20 (0.06)	18.56 (0.15)	42.46 (0.30)	86.32 (0.52)	174.34 (0.83)	388.82 (1.30)
7	7.96 (0.08)	1.55 (0.02)	1.76 (0.02)	8.45 (0.06)	12.14 (0.06)	18.80 (0.16)	43.22 (0.31)	88.64 (0.54)	176.29 (0.84)	389.03 (1.38)
8	8.52 (0.09)	1.56 (0.02)	1.85 (0.03)	9.14 (0.08)	13.02 (0.08)	20.24 (0.17)	45.94 (0.34)	93.64 (0.58)	184.50 (0.89)	400.83 (1.34)
9	9.07 (0.09)	1.69 (0.02)	1.91 (0.03)	9.37 (0.08)	13.79 (0.08)	21.80 (0.19)	49.75 (0.36)	100.29 (0.62)	195.51 (0.95)	411.40 (1.38)
10	8.05 (0.08)	1.46 (0.02)	1.71 (0.02)	8.54 (0.08)	12.29 (0.08)	19.38 (0.18)	44.67 (0.34)	92.66 (0.59)	181.46 (0.92)	386.48 (1.38)

Abbreviation: ASMR, age-specific mortality risk.

Associations between PM_2.5_ deciles at the census tract level and ASMR, with and without adjusting for SES parameters (ie, proportion of Black residents, proportion of residents aged 25 years or older with a college degree, median household income, proportion of residents below the federal poverty line, and population density) in the multilevel model were observed for all age groups (Table 4). The highest associations between the decile categorization of PM_2.5_ and ASMR were observed for individuals older than 45 years. Accounting for SES parameters attenuated the associations between PM_2.5_ concentrations and ASMR. However, even after this adjustment, the multilevel models showed a gradient of greater risk of death with an increase in the air pollution decile. The sensitivity analysis showed similar results when the census tract PM_2.5_ was analyzed as a continuous variable (eTable 5 in the Supplement). Statistically significant values were identified for individuals aged younger than 1 year (β = 0.105; 95% CI, 0.059-0.151), 5 to 14 years (β = 0.014; 95% CI, 0.001-0.027), and older than 45 years age groups (45-54 years: β = 0.631; 95% CI, 0.474-0.788; 55-64 years: β = 1.366; 95% CI, 1.119-1.613; 65-74 years: β = 2.073; 95% CI, 1.645-2.502; 75-84 years: β = 1.603; 95% CI, 0.803-2.403), where the β value was particularly high in the 3 oldest age groups. The findings in this study were robust when assessed using alternative periods of PM_2.5_ (eTable 6 in the Supplement).

**Table 4.** Crude and Adjusted Multilevel Regression Coefficient of Probability of Dying According to Census Tract Concentration of PM_2.5_ Deciles^a^

Age group, y	β coefficient (95% CI) by PM_2.5_ Decile^a^								
	2	3	4	5	6	7	8	9	10
**<1**									
Crude	0.03 (−0.22 to 0.28)	0.36 (0.09 to 0.63)	0.54 (0.26 to 0.82)	0.80 (0.52 to 1.08)	0.97 (0.68 to 1.26)	1.27 (0.98 to 1.57)	1.55 (1.25 to 1.86)	2.13 (1.81 to 2.44)	2.88 (2.51 to 3.26)
Adjusted	0.02 (−0.21 to 0.26)	0.04 (−0.22 to 0.31)	0.08 (−0.19 to 0.34)	0.14 (−0.14 to 0.42)	0.08 (−0.20 to 0.38)	0.28 (−0.02 to 0.58)	0.46 (0.14 to 0.77)	0.62 (0.28 to 0.96)	0.82 (0.43 to 1.21)
**1-4**									
Crude	−0.06 (−0.13 to 0.01)	−0.05 (−0.12 to 0.03)	−0.07 (−0.15 to 0.01)	−0.09 (−0.17 to −0.01)	−0.10 (−0.19 to −0.02)	−0.10 (−0.18 to −0.01)	−0.12 (−0.21 to −0.04)	0.04 (−0.04 to 0.14)	0.14 (0.04 to 0.25)
Adjusted	0.04 (−0.03 to 0.10)	0.05 (−0.02 to 0.12)	0.03 (−0.04 to 0.10)	0.02 (−0.06 to 0.10)	0.00 (−0.07 to 0.08)	0.02 (−0.06 to 0.10)	−0.00 (−0.09 to 0.08)	0.11 (0.02 to 0.20)	0.09 (−0.02 to 0.19)
**5-14**									
Crude	−0.04 (−0.11 to 0.04)	−0.05 (−0.13 to 0.03)	−0.03 (−0.12 to 0.05)	−0.02 (−0.11 to 0.07)	−0.12 (−0.21 to −0.03)	−0.10 (−0.19 to −0.01)	−0.09 (−0.19 to −0.00)	−0.02 (−0.11 to 0.08)	0.10 (−0.02 to 0.21)
Adjusted	0.09 (0.02 to 0.16)	0.08 (−0.00 to 0.16)	0.10 (0.02 to 0.18)	0.14 (0.05 to 0.22)	0.04 (−0.04 to 0.13)	0.08 (−0.01 to 0.17)	0.11 (0.02 to 0.20)	0.15 (0.05 to 0.25)	0.17 (0.05 to 0.28)
**15-24**									
Crude	−0.68 (−0.90 to −0.47)	−0.63 (−0.86 to −0.39)	−0.82 (−1.06 to −0.58)	−0.69 (−0.94 to −0.44)	−0.77 (−1.02 to −0.51)	−0.57 (−0.83 to −0.31)	−0.49 (−0.76 to −0.22)	−0.11 (−0.39 to 0.17)	0.67 (0.34 to 1.00)
Adjusted	−0.30 (−0.50 to −0.10)	−0.24 (−0.47 to −0.01)	−0.41 (−0.65 to −0.17)	−0.25 (−0.50 to −00)	−0.38 (−0.64 to −0.12)	−0.14 (−0.41 to 0.13)	−0.01 (−0.30 to 0.27)	0.17 (−0.13 to 0.48)	0.62 (0.26 to 0.98)
**25-34**									
Crude	−0.81 (−1.14 to −0.48)	−0.63 (−1.01 to −0.26)	−1.12 (−1.50 to −0.74)	−0.97 (−1.37 to −0.58)	−0.92 (−1.32 to −0.52)	−0.79 (−1.20 to −0.38)	−0.64 (−1.06 to −0.21)	0.05 (−0.39 to 0.50)	1.64 (1.12 to 2.16)
Adjusted	−0.28 (−0.59 to 0.03)	−0.08 (−0.44 to 0.27)	−0.52 (−0.89 to −0.16)	−0.32 (−0.71 to 0.06)	−0.39 (−0.79 to 0.01)	−0.29 (−0.71 to 0.12)	−0.05 (−0.49 to 0.39)	0.18 (−0.30 to 0.65)	1.16 (0.60 to 1.71)
**35-44**									
Crude	0.48 (−0.02 to 0.97)	2.04 (1.48 to 2.59)	2.77 (2.20 to 3.35)	3.64 (3.05 to 4.23)	4.77 (4.17 to 5.37)	5.93 (5.31 to 6.54)	7.16 (6.53 to 7.80)	9.56 (8.89 to 10.23)	12.91 (12.13 to 13.68)
Adjusted	−0.36 (−0.78 to 0.06)	−0.24 (−0.72 to 0.24)	−0.47 (−0.97 to 0.03)	−0.35 (−0.88 to 0.18)	−0.26 (−0.81 to 0.28)	0.04 (−0.53 to 0.61)	0.52 (−0.08 to 1.12)	0.86 (0.21 to 1.52)	1.92 (1.16 to 2.68)
**45-54**									
Crude	4.38 (3.45 to 5.32)	9.32 (8.27 to 10.36)	12.34 (11.25 to 13.42)	15.18 (14.06 to 16.30)	18.58 (17.44 to 19.72)	21.74 (20.57 to 22.90)	25.10 (23.90 to 26.31)	31.94 (30.67 to 33.21)	39.55 (38.09 to 41.01)
Adjusted	0.91 (019 to 1.16)	1.48 (0.67 to 2.29)	1.35 (0.50 to 2.20)	1.73 (0.83 to 2.62)	2.20 (1.27 to 3.12)	2.92 (1.95 to 3.88)	3.82 (2.80 to 4.85)	5.13 (4.02 to 6.23)	6.53 (5.24 to 7.81)
**55-64**									
Crude	9.82 (8.24 to 11.40)	18.49 (16.71 to 20.26)	24.66 (22.82 to 26.49)	29.18 (27.29 to 31.08)	35.32 (33.39 to 37.24)	40.78 (38.82 to 42.75)	46.84 (44.81 to 48.88)	59.13 (56.98 to 61.28)	72.49 (70.02 to 74.97)
Adjusted	3.23 (2.09 to 4.38)	3.87 (2.58 to 5.17)	4.43 (3.08 to 5.78)	4.51 (3.10 to 5.93)	5.29 (3.82 to 6.76)	6.69 (5.16 to 8.22)	8.40 (6.78 to 10.01)	10.55 (8.80 to 12.30)	12.12 (10.09 to 14.15)
**65-74**									
Crude	15.53 (13.00 to 18.06)	29.05 (26.22 to 18.06)	37.48 (34.56 to 40.40)	43.12 (40.11 to 46.13)	50.94 (47.87 to 54.00)	56.44 (53.31 to 59.57)	64.55 (61.32 to 67.78)	80.90 (77.49 to 84.31)	97.52 (93.56 to 101.48)
Adjusted	5.50 (3.48 to 7.51)	7.57 (5.30 to 9.83)	8.73 (6.37 to 11.08)	8.43 (5.95 to 10.90)	9.28 (6.70 to 11.85)	9.73 (7.05 to 12.40)	12.26 (9.44 to 15.09)	15.31 (12.27 to 18.36)	15.58 (12.05 to 19.11)
**75-84**									
Crude	21.86 (17.80 to 25.92)	32.75 (28.26 to 37.24)	36.51 (31.94 to 41.08)	41.77 (37.08 to 46.47)	47.02 (42.25 to 51.80)	47.89 (43.03 to 52.75)	55.21 (50.21 to 60.22)	66.78 (61.50 to 72.06)	72.86 (66.68 to 79.04)
Adjusted	10.42 (6.54 to 14.29)	11.83 (7.49 to 16.17)	10.33 (5.84 to 14.82)	10.30 (5.58 to 15.01)	10.56 (5.66 to 15.46)	7.99 (2.90 to 13.07)	11.62 (6.27 to 16.97)	13.22 (7.46 to 18.98)	7.89 (1.24 to 14.54)

Abbreviation: PM_2.5_, particulate matter 2.5 μm or smaller.

^a^ Adjusted by census tract proportion of Black residents, proportion of people aged 25 years or older with a college degree, median household income, proportion of residents below the federal poverty line, and population density.

Analyses stratified by SES showed that the highest β values were observed in census tracts with the lowest median household income quintile, lowest quintile of college-educated residents older than 25 years, the highest quintile of the proportion of Black residents, highest quintile of residents below the federal poverty line, and highest population density (eTables 7-11 in the Supplement).

## Discussion

This cross-sectional study presents a systematic and comprehensive analysis of the association among PM_2.5_ concentration, SES, and ASMR across census tracts in the US. We report 6 key findings. First, ASMR and exposure to PM_2.5_ varied substantially across the US census tracts. Second, census tracts accounted for most of the total variability in ASMR. Third, SES and air pollution variables were associated with much of the variance in ASMR observed, mainly at the state and county levels. At census tract–level, most of the variance in ASMR remained unexplained by the observable SES and air pollution variables, especially for individuals aged younger than 35 years. Fourth, census tracts with a higher proportion of Black residents, a higher population density, a higher proportion of residents living in poverty, and a smaller share of residents with a college education had higher PM_2.5_ concentrations. Fifth, the fully adjusted multilevel models showed a robust association between air pollution and ASMR across all age groups, particularly in the groups older than 45 years. Sixth, the risk of ASMR associated with PM_2.5_ was higher in the most underprivileged census tracts.

The robust association between long-term exposure to PM_2.5_ concentrations and the risk of mortality is consistent with epidemiological evidence.^7,25^ Different pathways may explain the increased risk of mortality among people exposed to higher concentrations of PM_2.5_. PM_2.5_ particles have the ability to penetrate the respiratory system and directly enter the bloodstream and specific organs, aggravating local oxidative stress and inflammation. Inflammation-related cytokine genes are stimulated, and inflammatory injuries may occur. Additionally, inflammatory cells and cytokines can damage lung cells synergistically.^26^ According to a review by Du et al,^27^ such a systemic inflammatory process is a risk factor associated with atherosclerosis progression, and the cascade of events associated with it may exacerbate myocardial ischemia. Other mechanisms through which PM_2.5_ damages the body include cell injuries from free-radical peroxidation and imbalanced intracellular calcium homeostasis.^26^ A 2008 study^28^ reported that PM_2.5_ particles may damage DNA and suppress DNA repair. Given the biological outcomes associated with exposure to air pollutions, it is expected that the clinical outcomes among older adults would be more acute that those among younger people, considering the potential for longer exposures to pollutants and many other health hazards, which may have synergic action, and less capacity for the body to biologically respond to the challenges imposed by pollution in older adults. Similarly, individuals with lower SES have a higher burden of diseases, more body cell and tissue damage, and more obstacles that limit access to health services, a healthy diet, and healthy habits and behaviors.

Regarding the spatial patterning in the exposure response between long-term exposure to PM_2.5_ and the risk of mortality, there are a number of plausible explanations for the bulk of variation occurring at the census tract level. First, regionally varying and neighborhood-level contextual parameters, such as traffic, composition of PM_2.5_, age of homes, and their position relative to large roads, may enhance exposure gradients within a particular county or state.^29^ Other spatially structured individual factors may further lead to increased risk of the adverse health outcomes associated with air pollution. Spatially varying individual-level factors, such as psychosocial, occupational, and nutritional risks, are also important spatial determinants of the associations of PM_2.5_ with the risk of mortality. These observations suggest that the associations of PM_2.5_ with mortality may be modified by location. The important roles of SES demonstrate that policies aimed at reducing pollutants in the US should consider not only the overall emission reductions but also racial, spatial, and socioeconomic inequalities.

The inequality in exposure to PM_2.5_ associated with SES observed in our study is consistent with that observed in previous studies that also found that Black individuals and individuals with low incomes had the highest exposures.^30,31,32^ This finding has important environmental justice implications, since inequalities in exposure to PM_2.5_ levels are increasing.^17,33^ Different theories have tried to explain such inequalities. Economically, industries look for cheap land and cheap labor, and these are more easily found in poorer regions.^34^ Moreover, people who are wealthier tend to move out from neighborhoods where pollutant-emitting industries are situated. In addition, more affluent regions have more economic and political resources to oppose the establishment of such industries in their vicinity.^34^ Racial segregation in the occupation of different US territories and the racism present in past and present public policies that increase the risk of exposure to environmental risk factors for the Black population in the US must also be considered.^35^ Such circumstances, in part, explain the higher risk of mortality and lower life expectancy among Black individuals in the US. Finally, the political decentralization in the United States may lead to greater inequality in social and health outcomes, since public policies can express different views of fairness and solidarity of each locality.^36,37^ Focusing on health services, there are important inequalities in access to medical care across areas of the US,^38^ including a higher proportion of people without health insurance in regions with higher levels of unemployment and poverty.^39^

What we can observe from these findings is that public policies to improve air quality in the US need to be equitable. The social, labor, geographic, and economic contexts in which populations live need to be considered when designing actions to combat air pollution. As SES is associated with risk of exposure to environmental hazards, improving air quality involves improving the social determinants of health.

### Limitations

This study has several limitations that need to be considered when analyzing the results. First, it was a cross-sectional study that used spatially aggregated data. Therefore, we cannot establish causal relationships between PM_2.5_ exposure and ASMR. We were unable to adjust for individual-level behavioral and biological confounders in our models. Second, the geographic units used were politically and administratively defined and may not accurately represent the exposure of the inhabitants of a given region to the variables analyzed. Third, not all American census tracts were analyzed. Those who didn’t meet methodological criteria were excluded. Fourth, the estimated PM_2.5_ concentrations have some exposure measurement error; however, the estimation models have good estimation accuracy. Fifth, to estimate the risk of mortality in each age group in some census tracts, it was necessary to calculate the probability of death owing to missing values. Such calculations were based on the combination of socioeconomic and demographic characteristics of the census tracts; this may have affected the estimated associations.^20^ We could not exclude census tracts with long-term care facilities for older adults that may have an inflated number of deaths, which may have inflated the variance at the census tract level. Hence, it is advisable to be cautious with population estimates made using small populations and territorial content data, such as census tracts.

## Conclusions

The findings of this cross-sectional study suggest that efforts to increase in life expectancy in the US in the future should involve, in part, lower exposure of its population to air pollution. The greater risk of mortality in regions with higher levels of PM_2.5_ across all age groups suggests that improving air quality is urgently needed in the US. Moreover, the observation that PM_2.5_ is unevenly distributed in the US, with higher concentrations in the most underprivileged regions, suggests the need for more equitable policies on overall air improvement.

## Notes

Supplement. **eFigure 1.** Definition of the Probability of Death Between 2 Exact Ages **eFigure 2.** Flowchart of the Multilevel Data Structure and Covariates **eFigure 3.** Geographic Distribution of Probability of Dying Among Children Younger Than 1 Year **eFigure 4.** Geographic Distribution of Probability of Dying in the Group Aged 1-4 Years **eFigure 5.** Geographic Distribution of Probability of Dying in the Group Aged 5-14 Years **eFigure 6.** Geographic Distribution of Probability of Dying in the Group Aged 15-24 Years **eFigure 7.** Geographic Distribution of Probability of Dying in the Group Aged 25-34 Years **eFigure 8.** Geographic Distribution of Probability of Dying in the Group Aged 35-44 Years **eFigure 9.** Geographic Distribution of Probability of Dying in the Group Aged 45-54 Years **eFigure 10.** Geographic Distribution of Probability of Dying in the Group Aged 55-64 Years **eFigure 11.** Geographic Distribution of Probability of Dying in the Group Aged 65-74 Years **eFigure 12.** Geographic Distribution of Probability of Dying in the Group Aged 75-84 Years **eFigure 13.** Geographic Distribution of Proportion of People Aged 25 Years or Older With a College Degree **eFigure 14.** Geographic Distribution of Proportion of Residents Below the Federal Poverty Line **eFigure 15.** Geographic Distribution of Median Household Income **eFigure 16.** Geographic Distribution of Population Density **eFigure 17.** Geographic Distribution of Proportion of Black Residents **eTable 1.** Variance in Probability of Dying by Age Groups and Variance Change From Null Model When Adjusted by Census Tract Concentration PM_2.5_ and Socioeconomic and Demographic Variables **eTable 2.** Variance in Probability of Dying by Age Groups and Variance Change From Null Model When Adjusted by Census Tract Socioeconomic and Demographic Variables **eTable 3.** Variance in Probability of Dying by Age Groups, and Percentage of Variance Change From the Null Model When Adjusted by Census Tract Concentration of PM_2.5_ **eTable 4.** Crude and Adjusted Multilevel Regression Coefficient of PM_2.5_ and Socioeconomic and Demographic Variables **eTable 5.** Crude and Adjusted Multilevel Regression Coefficient of Probability of Dying (×1000) According to Census Tract PM_2.5_ as a Continuous Variable **eTable 6.** Crude and Adjusted Multilevel Regression Coefficient of Probability of Dying According to Census Tract Concentration of PM_2.5_ Deciles **eTable 7.** Multilevel Regression Coefficient of Probability of Dying According to Census Tract Concentration of PM_2.5_ Deciles and Median Household Income Quintiles **eTable 8.** Multilevel Regression Coefficient of Probability of Dying According to Census Tract Concentration of PM_2.5_ Deciles and Proportion of Black Residents **eTable 9.** Multilevel Regression Coefficient of Probability of Dying According to Census Tract Concentration of PM_2.5_ Deciles and Share of Residents Below the Federal Poverty Line Quintiles **eTable 10.** Multilevel Regression Coefficient of Probability of Dying According to Census Tract Concentration of PM_2.5_ Deciles and Population Density Quintiles **eTable 11.** Multilevel Regression Coefficient of Probability of Dying According to Census Tract Concentration of PM_2.5_ Deciles and Quintiles of the Proportion of People Aged 25 Years or Older With a College Degree
