# Supplementary material for: Air Pollution, Socioeconomic Status, and Age-Specific Mortality Risk in the United States
Source: JAMA Netw Open. 2022 May 24;5(5):e2213540. doi: 10.1001/jamanetworkopen.2022.13540 (PMC9131742; doi:10.1001/jamanetworkopen.2022.13540)
Supplement: Supplement. — eFigure 1. Definition of the Probability of Death Between 2 Exact Ages eFigure 2. Flowchart of the Multilevel Data Structure and Covariates eFigure 3. Geographic Distribution of Probability of Dying Among Children Younger Than 1 Year eFigure 4. Geographic Distribution of Probability of Dying in the Group Aged 1-4 Years eFigure 5. Geographic Distribution of Probability of Dying in the Group Aged 5-14 Years eFigure 6. Geographic Distribution of Probability of Dying in the Group Aged 15-24 Years eFigure 7. Geographic Distribution of Probability of Dying in the Group Aged 25-34 Years eFigure 8. Geographic Distribution of Probability of Dying in the Group Aged 35-44 Years eFigure 9. Geographic Distribution of Probability of Dying in the Group Aged 45-54 Years eFigure 10. Geographic Distribution of Probability of Dying in the Group Aged 55-64 Years eFigure 11. Geographic Distribution of Probability of Dying in the Group Aged 65-74 Years eFigure 12. Geographic Distribution of Probability of Dying in the Group Aged 75-84 Years eFigure 13. Geographic Distribution of Proportion of People Aged 25 Years or Older With a College Degree eFigure 14. Geographic Distribution of Proportion of Residents Below the Federal Poverty Line eFigure 15. Geographic Distribution of Median Household Income eFigure 16. Geographic Distribution of Population Density eFigure 17. Geographic Distribution of Proportion of Black Residents eTable 1. Variance in Probability of Dying by Age Groups and Variance Change From Null Model When Adjusted by Census Tract Concentration PM2.5 and Socioeconomic and Demographic Variables eTable 2. Variance in Probability of Dying by Age Groups and Variance Change From Null Model When Adjusted by Census Tract Socioeconomic and Demographic Variables eTable 3. Variance in Probability of Dying by Age Groups, and Percentage of Variance Change From the Null Model When Adjusted by Census Tract Concentration of PM2.5 eTable 4. Crude and Adjusted Multilevel Regression Coefficien [file jamanetwopen-e2213540-s001.pdf]

Supplemental Online Content

Boing AF, deSouza P, Boing AC, Kim R, Subramanian SV. Air pollution, socioeconomic status, and age-specific mortality risk in the United States. *JAMA Netw Open*. 2022;5(5):e2213540. doi:10.1001/jamanetworkopen.2022.13540

eFigure 1. Definition of the Probability of Death Between 2 Exact Ages
eFigure 2. Flowchart of the Multilevel Data Structure and Covariates
eFigure 3. Geographic Distribution of Probability of Dying Among Children Younger Than 1 Year
eFigure 4. Geographic Distribution of Probability of Dying in the Group Aged 1-4 Years
eFigure 5. Geographic Distribution of Probability of Dying in the Group Aged 5-14 Years
eFigure 6. Geographic Distribution of Probability of Dying in the Group Aged 15-24 Years
eFigure 7. Geographic Distribution of Probability of Dying in the Group Aged 25-34 Years
eFigure 8. Geographic Distribution of Probability of Dying in the Group Aged 35-44 Years
eFigure 9. Geographic Distribution of Probability of Dying in the Group Aged 45-54 Years
eFigure 10. Geographic Distribution of Probability of Dying in the Group Aged 55-64 Years
eFigure 11. Geographic Distribution of Probability of Dying in the Group Aged 65-74 Years
eFigure 12. Geographic Distribution of Probability of Dying in the Group Aged 75-84 Years
eFigure 13. Geographic Distribution of Proportion of People Aged 25 Years or Older With a College Degree
eFigure 14. Geographic Distribution of Proportion of Residents Below the Federal Poverty Line
eFigure 15. Geographic Distribution of Median Household Income
eFigure 16. Geographic Distribution of Population Density
eFigure 17. Geographic Distribution of Proportion of Black Residents
eTable 1. Variance in Probability of Dying by Age Groups and Variance Change From Null Model When Adjusted by Census Tract Concentration $PM_{2.5}$ and Socioeconomic and Demographic Variables
eTable 2. Variance in Probability of Dying by Age Groups and Variance Change From Null Model When Adjusted by Census Tract Socioeconomic and Demographic Variables
eTable 3. Variance in Probability of Dying by Age Groups, and Percentage of Variance Change From the Null Model When Adjusted by Census Tract Concentration of $PM_{2.5}$

© 2022 Boing AF et al. *JAMA Network Open.*

eTable 4. Crude and Adjusted Multilevel Regression Coefficient of $PM_{2.5}$ and Socioeconomic and Demographic Variables
eTable 5. Crude and Adjusted Multilevel Regression Coefficient of Probability of Dying (×1000) According to Census Tract $PM_{2.5}$ as a Continuous Variable
eTable 6. Crude and Adjusted Multilevel Regression Coefficient of Probability of Dying According to Census Tract Concentration of $PM_{2.5}$ Deciles
eTable 7. Multilevel Regression Coefficient of Probability of Dying According to Census Tract Concentration of $PM_{2.5}$ Deciles and Median Household Income Quintiles
eTable 8. Multilevel Regression Coefficient of Probability of Dying According to Census Tract Concentration of $PM_{2.5}$ Deciles and Proportion of Black Residents
eTable 9. Multilevel Regression Coefficient of Probability of Dying According to Census Tract Concentration of $PM_{2.5}$ Deciles and Share of Residents Below the Federal Poverty Line Quintiles
eTable 10. Multilevel Regression Coefficient of Probability of Dying According to Census Tract Concentration of $PM_{2.5}$ Deciles and Population Density Quintiles
eTable 11. Multilevel Regression Coefficient of Probability of Dying According to Census Tract Concentration of $PM_{2.5}$ Deciles and Quintiles of the Proportion of People Aged 25 Years or Older With a College Degree

This supplemental material has been provided by the authors to give readers additional information about their work.

© 2022 Boing AF et al. *JAMA Network Open.*

eFigure 1. Definition of the Probability of Death Between 2 Exact Ages

$$ {}_nq_x = \frac{n_x * {}_nM_x}{1 + (1 - a_x) * n_x * {}_nM_x} $$

Note: ${}_nM_x$ is the age-specific period death rate, a_x is the fraction of life lived by those who died in the considered age interval, and n_x is the size of the age interval (years). Arias et al.[22]

© 2022 Boing AF et al. *JAMA Network Open.*

eFigure 2. Flowchart of the Multilevel Data Structure and Covariates

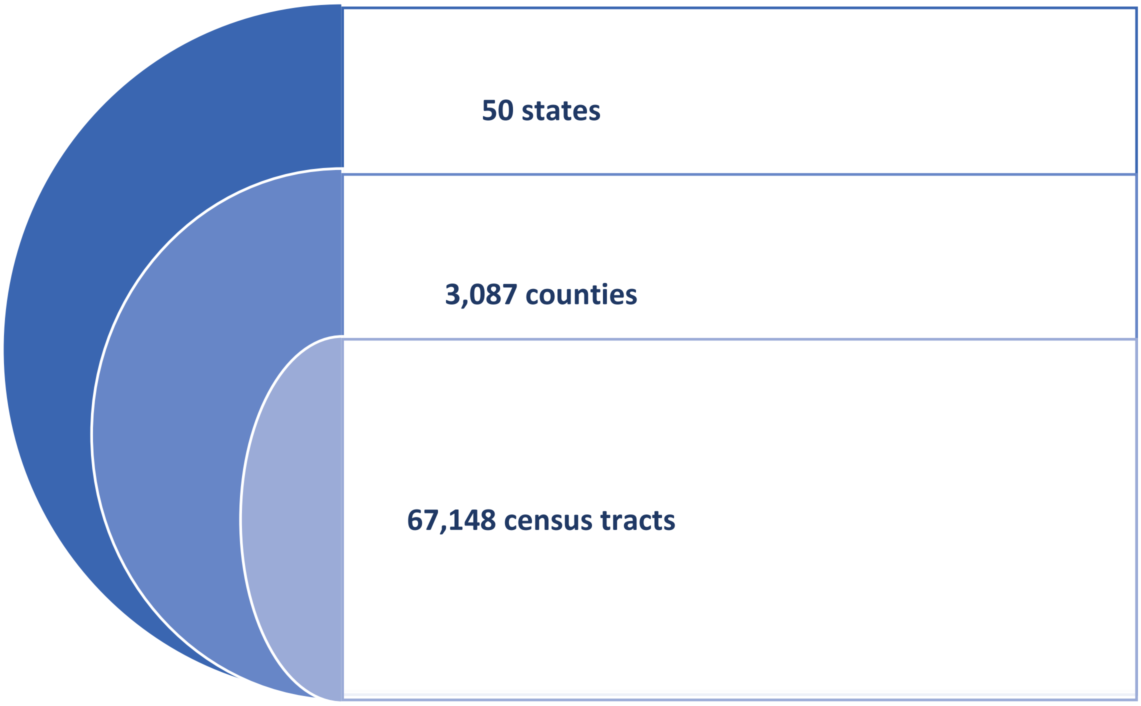

© 2022 Boing AF et al. *JAMA Network Open*.

eFigure 3. Geographic Distribution of Probability of Dying Among Children Younger Than 1 Year

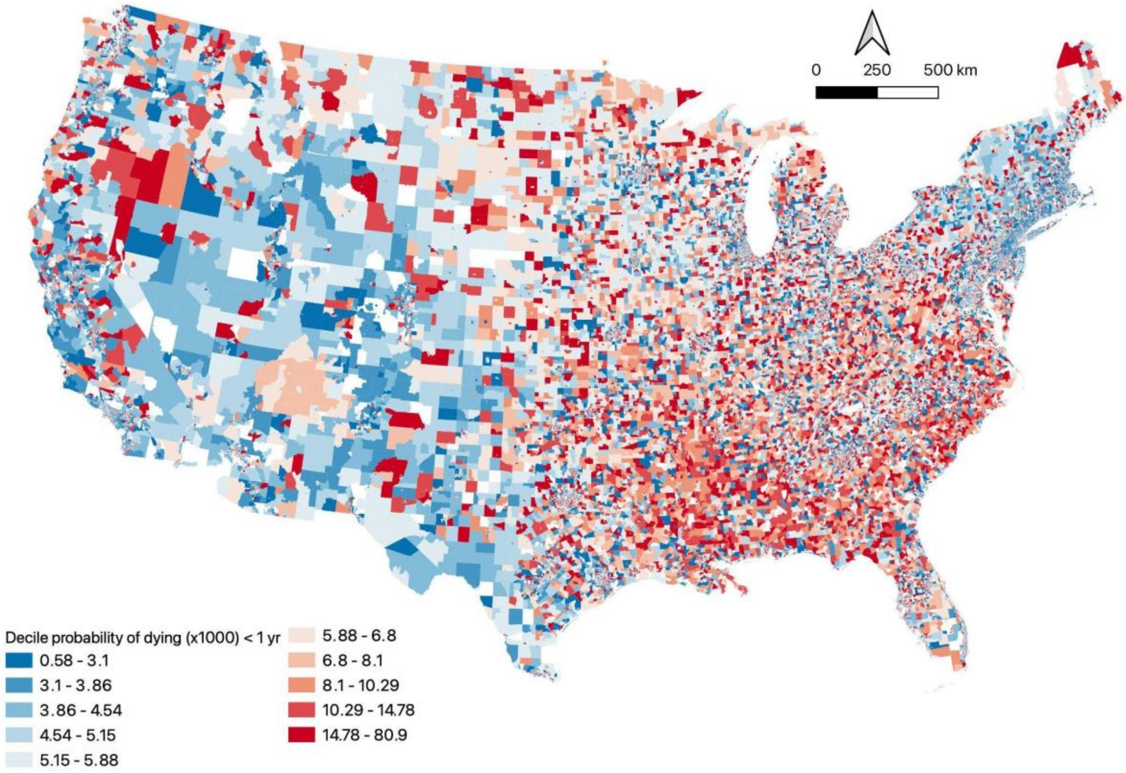

© 2022 Boing AF et al. *JAMA Network Open.*

eFigure 4. Geographic Distribution of Probability of Dying in the Group Aged 1-4 Years

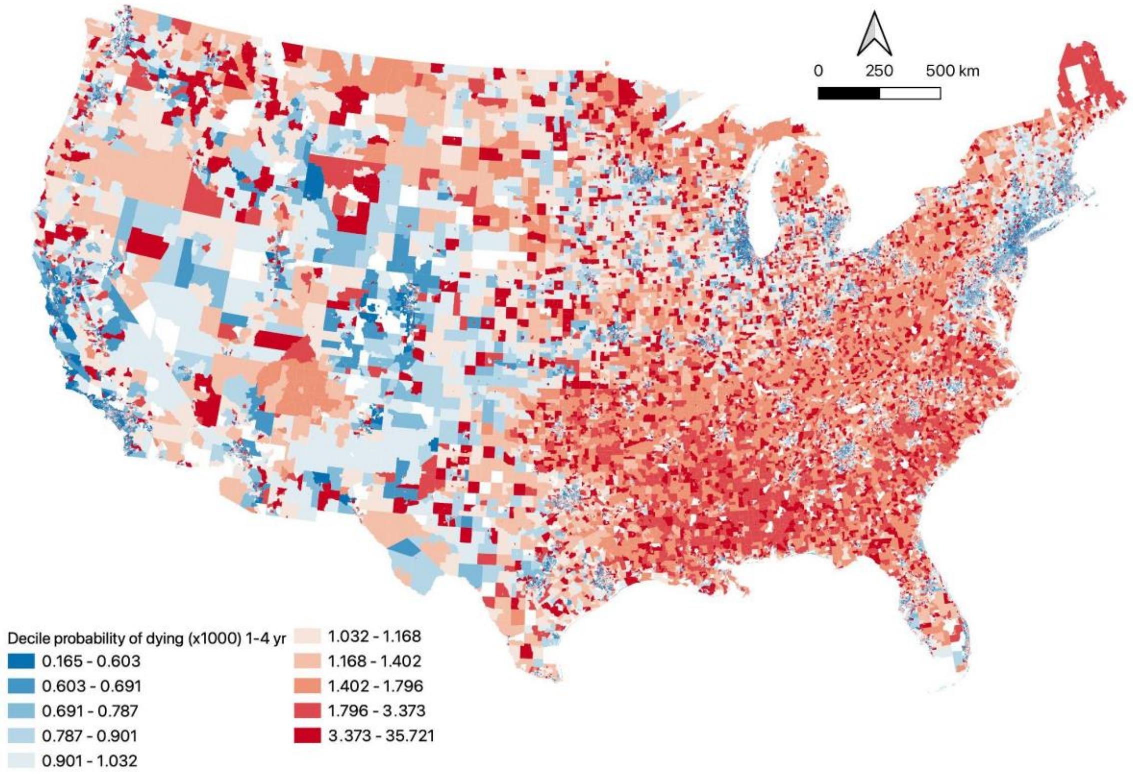

© 2022 Boing AF et al. *JAMA Network Open.*

eFigure 5. Geographic Distribution of Probability of Dying in the Group Aged 5-14 Years

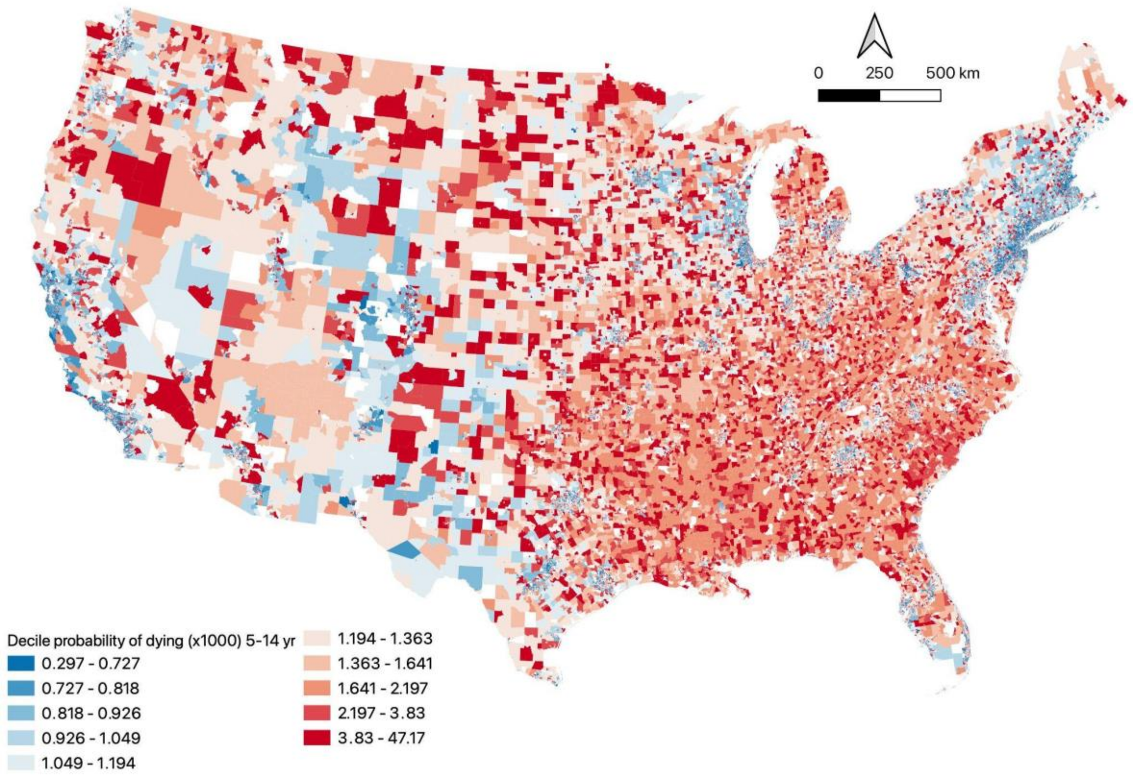

© 2022 Boing AF et al. *JAMA Network Open.*

eFigure 6. Geographic Distribution of Probability of Dying in the Group Aged 15-24 Years

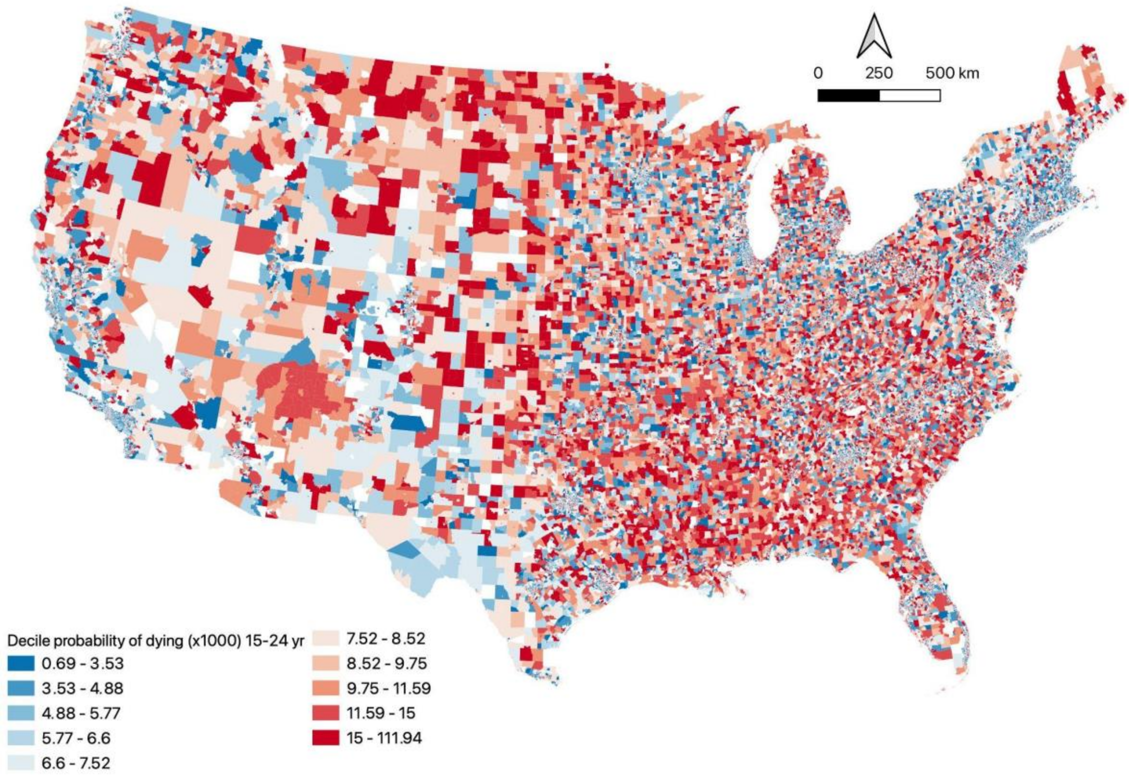

© 2022 Boing AF et al. *JAMA Network Open.*

eFigure 7. Geographic Distribution of Probability of Dying in the Group Aged 25-34 Years

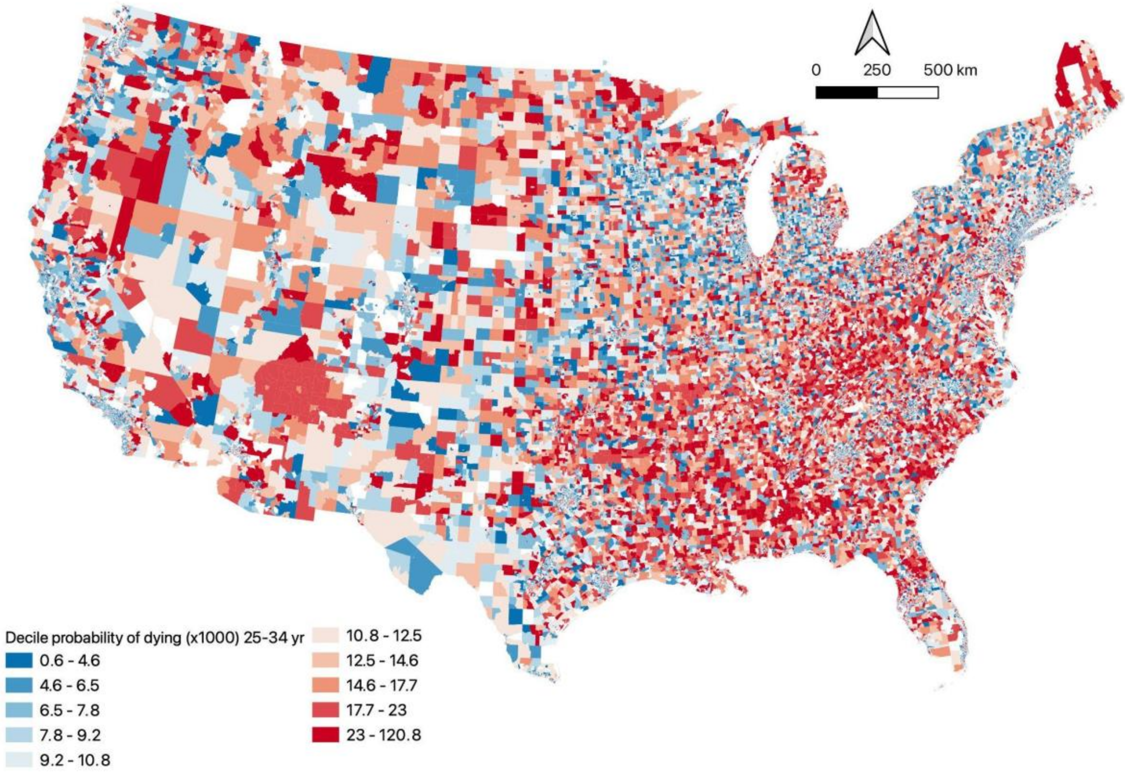

© 2022 Boing AF et al. *JAMA Network Open.*

eFigure 8. Geographic Distribution of Probability of Dying in the Group Aged 35-44 Years

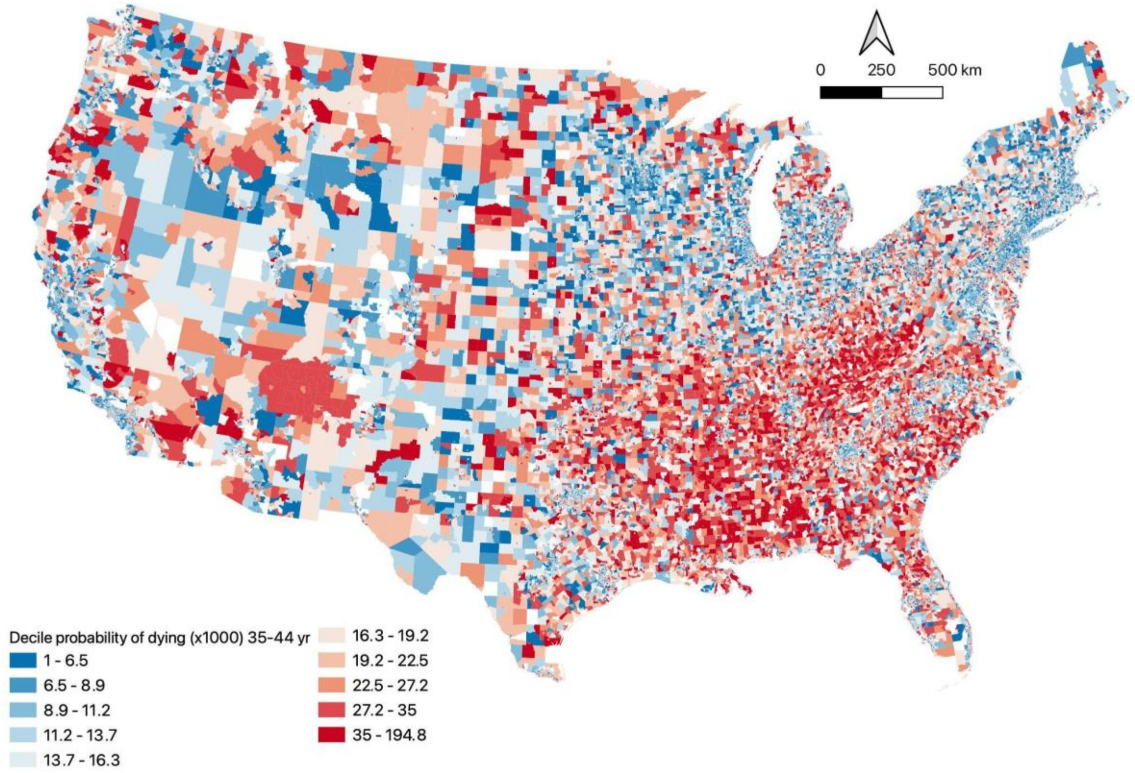

© 2022 Boing AF et al. *JAMA Network Open.*

eFigure 9. Geographic Distribution of Probability of Dying in the Group Aged 45-54 Years

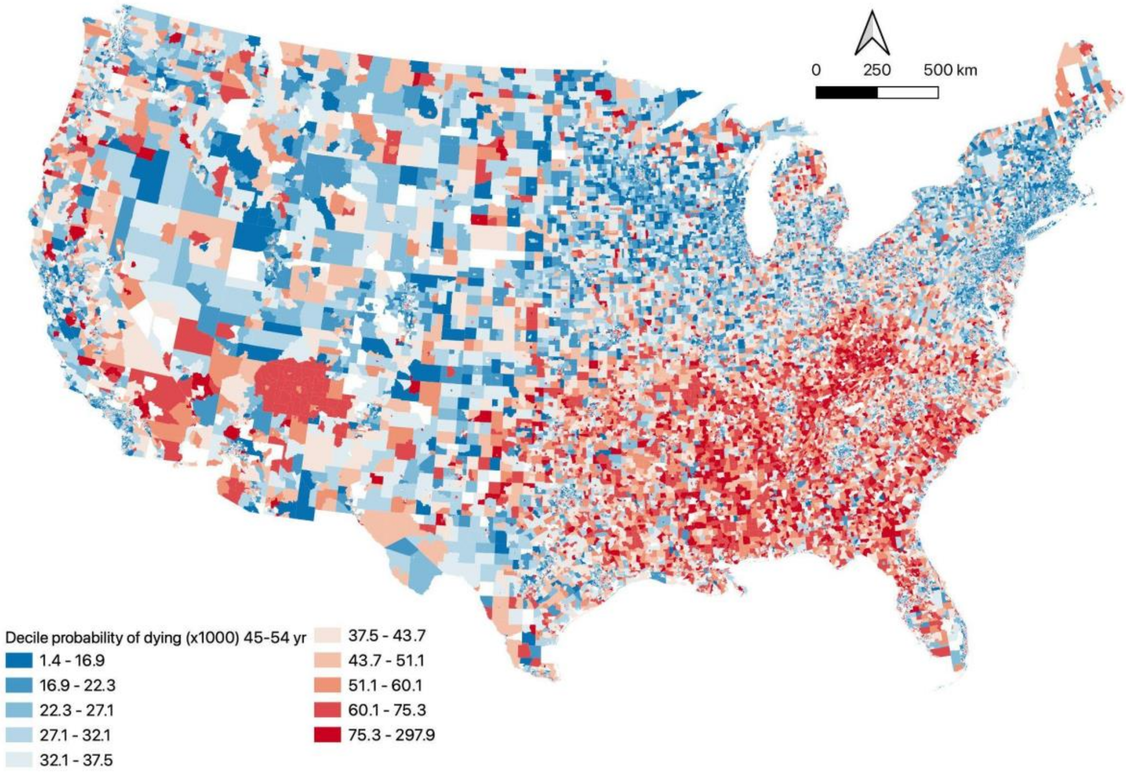

© 2022 Boing AF et al. *JAMA Network Open.*

eFigure 10. Geographic Distribution of Probability of Dying in the Group Aged 55-64 Years

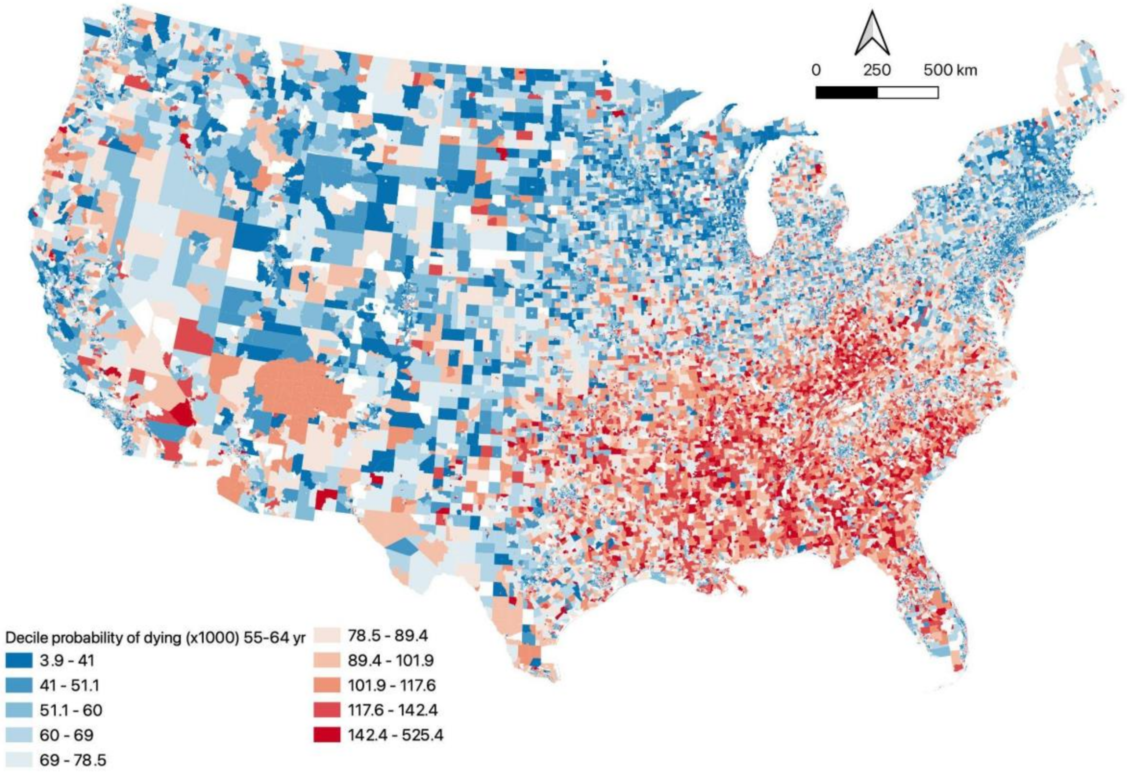

© 2022 Boing AF et al. *JAMA Network Open.*

eFigure 11. Geographic Distribution of Probability of Dying in the Group Aged 65-74 Years

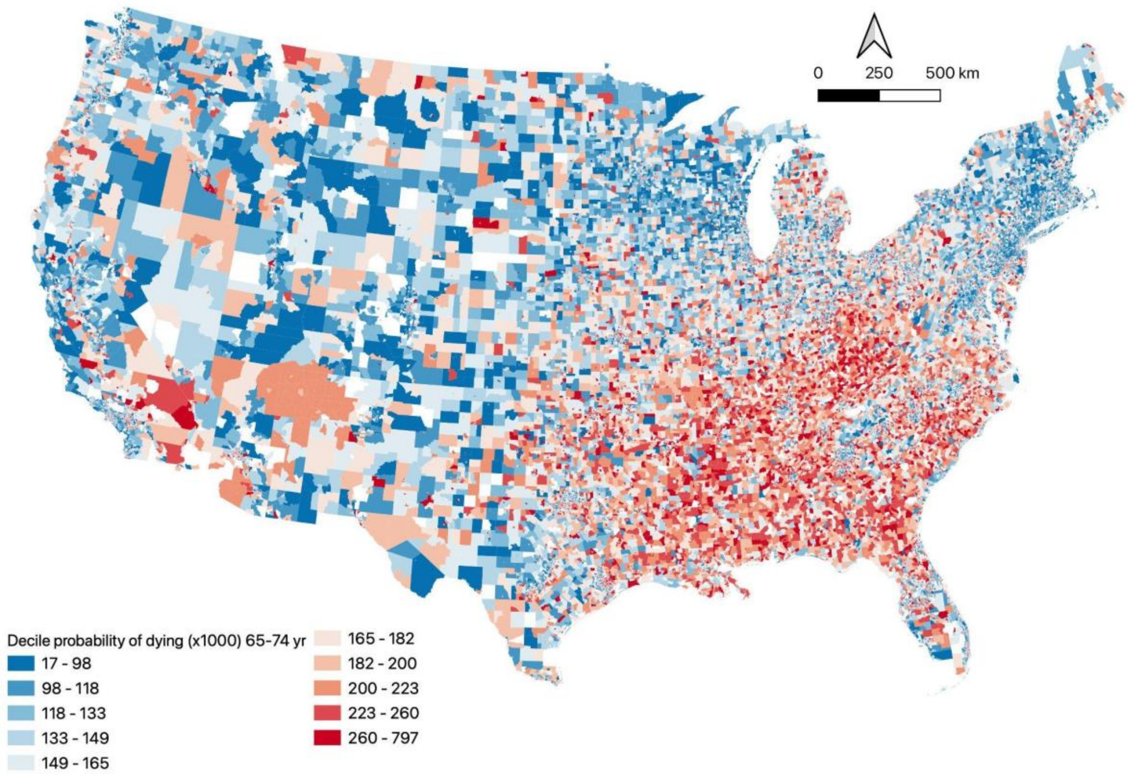

© 2022 Boing AF et al. *JAMA Network Open.*

eFigure 12. Geographic Distribution of Probability of Dying in the Group Aged 75-84 Years

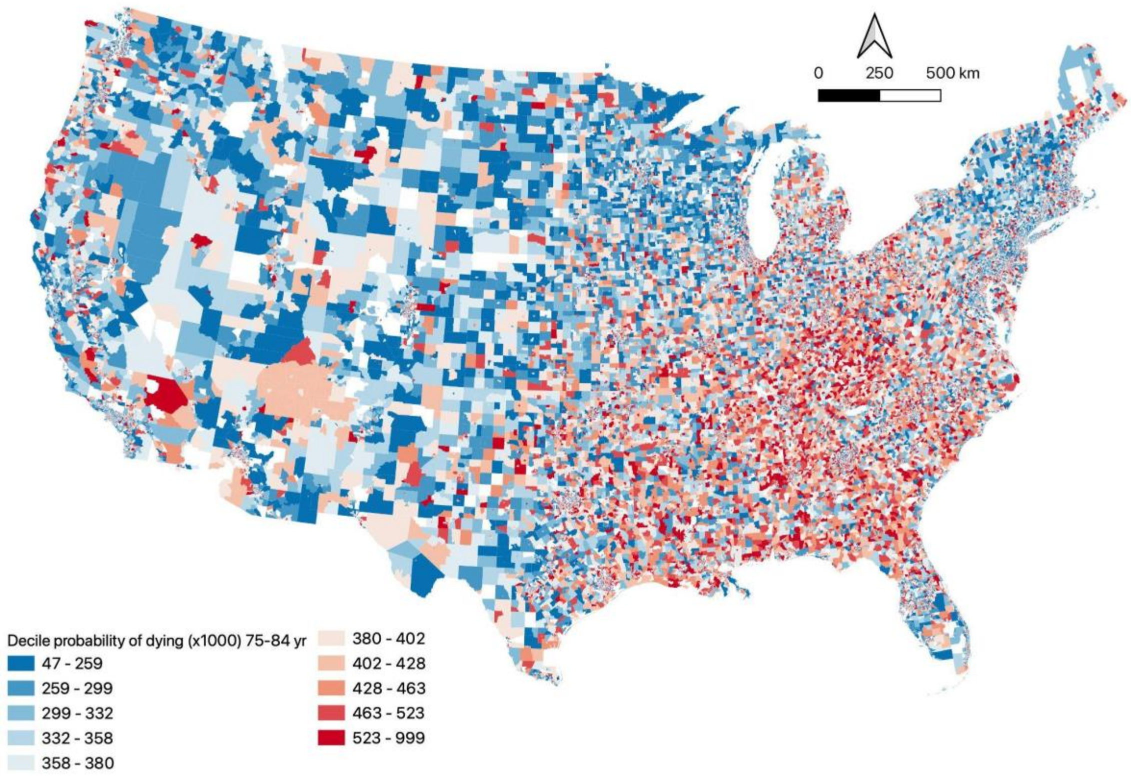

© 2022 Boing AF et al. *JAMA Network Open.*

eFigure 13. Geographic Distribution of Proportion of People Aged 25 Years or Older With a College Degree

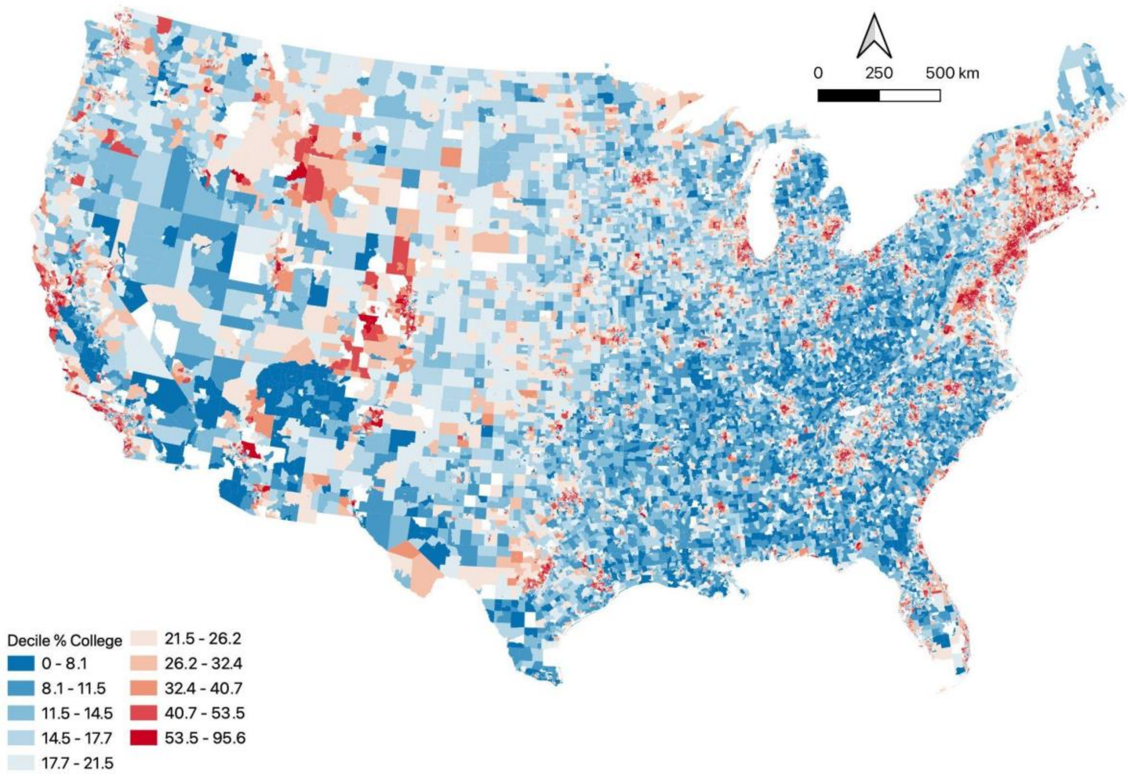

© 2022 Boing AF et al. *JAMA Network Open.*

eFigure 14. Geographic Distribution of Proportion of Residents Below the Federal Poverty Line

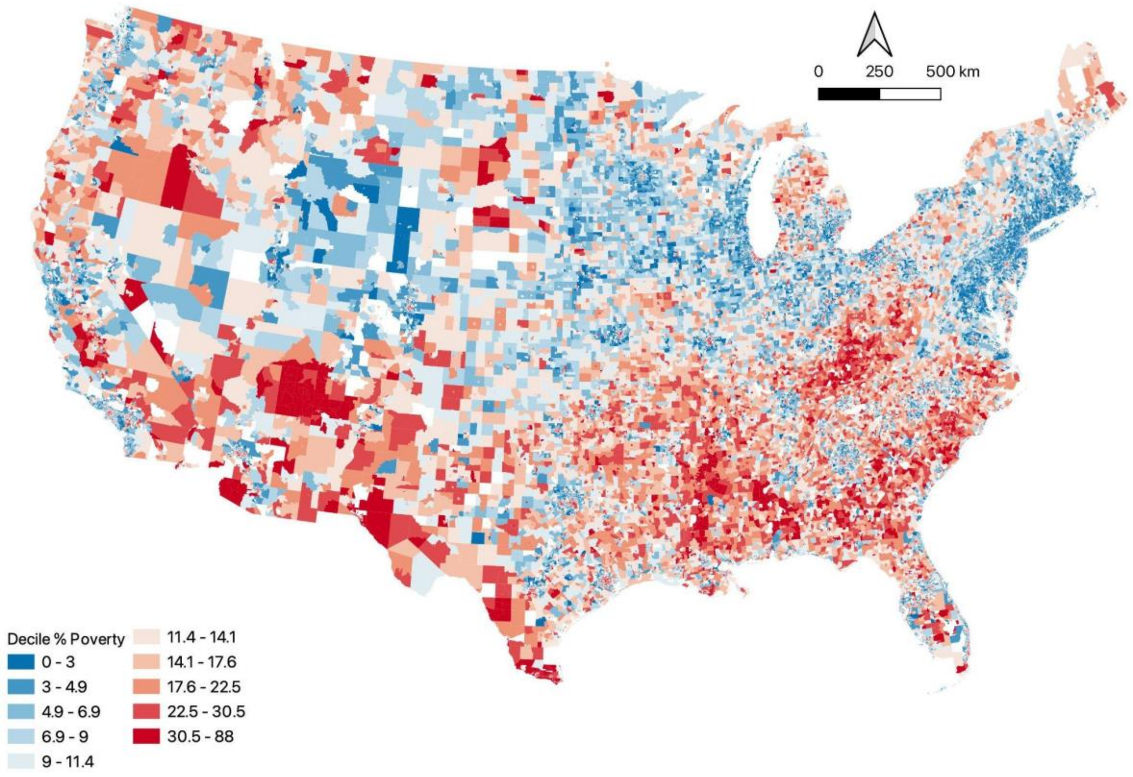

© 2022 Boing AF et al. *JAMA Network Open.*

eFigure 15. Geographic Distribution of Median Household Income

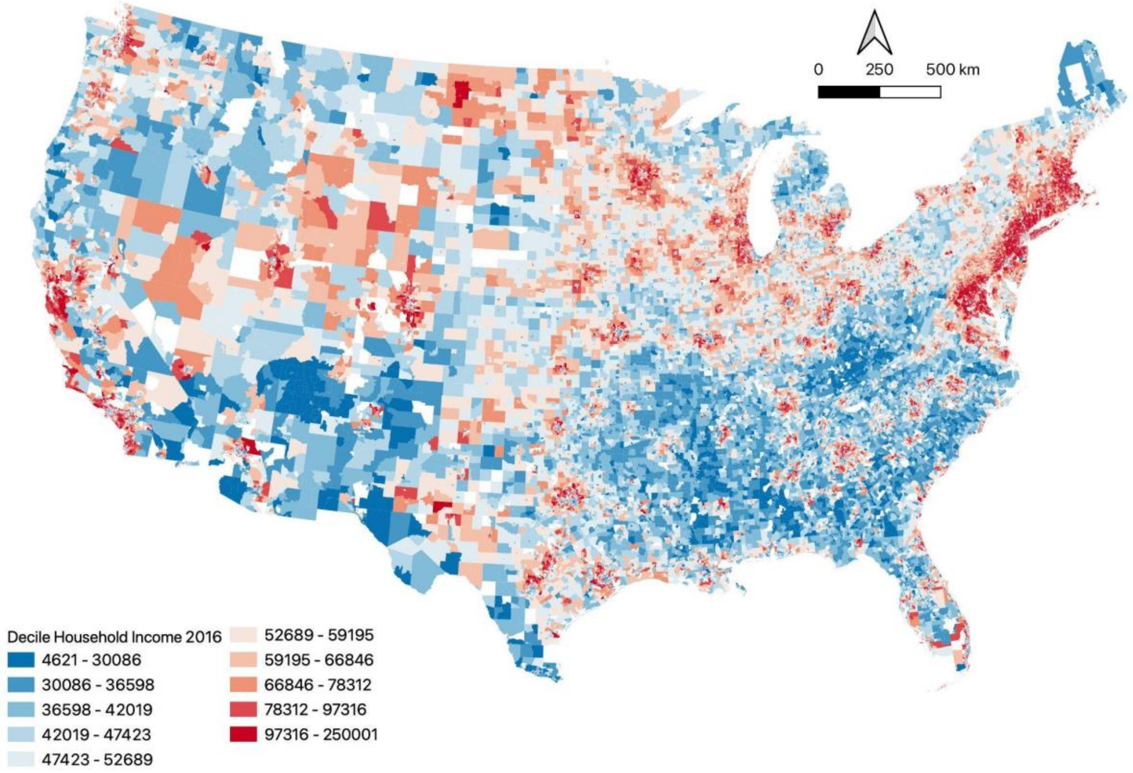

© 2022 Boing AF et al. *JAMA Network Open.*

eFigure 16. Geographic Distribution of Population Density

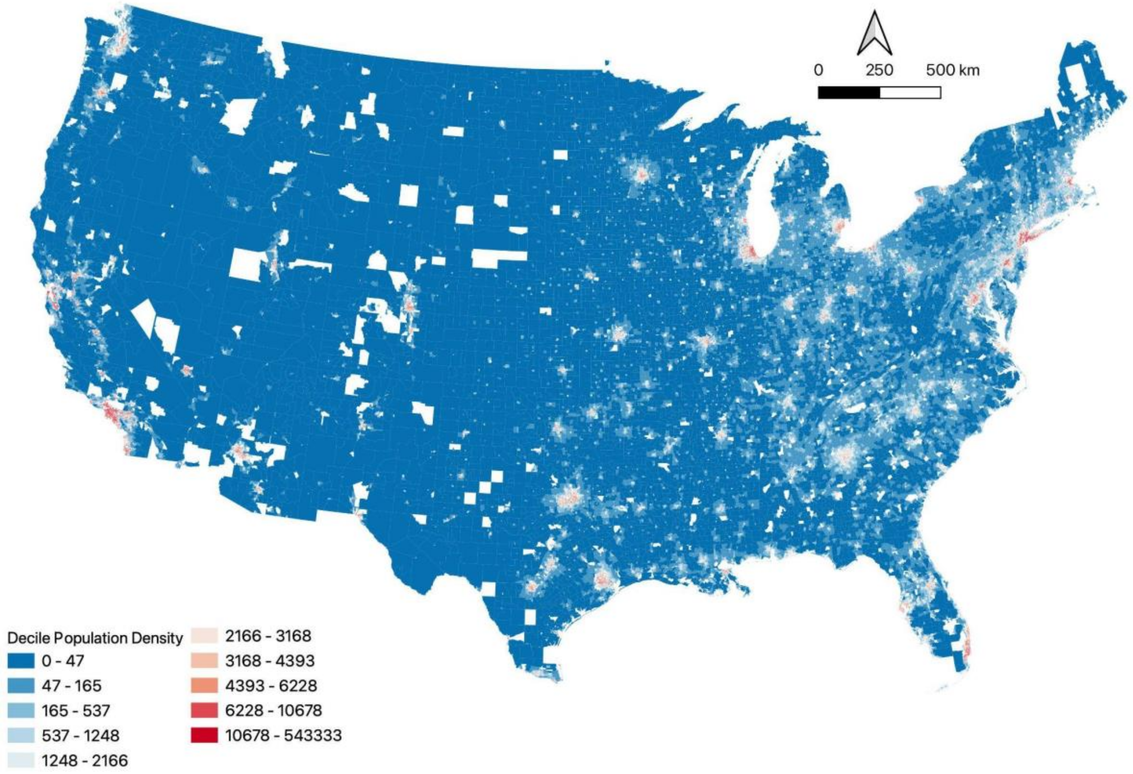

© 2022 Boing AF et al. *JAMA Network Open.*

eFigure 17. Geographic Distribution of Proportion of Black Residents

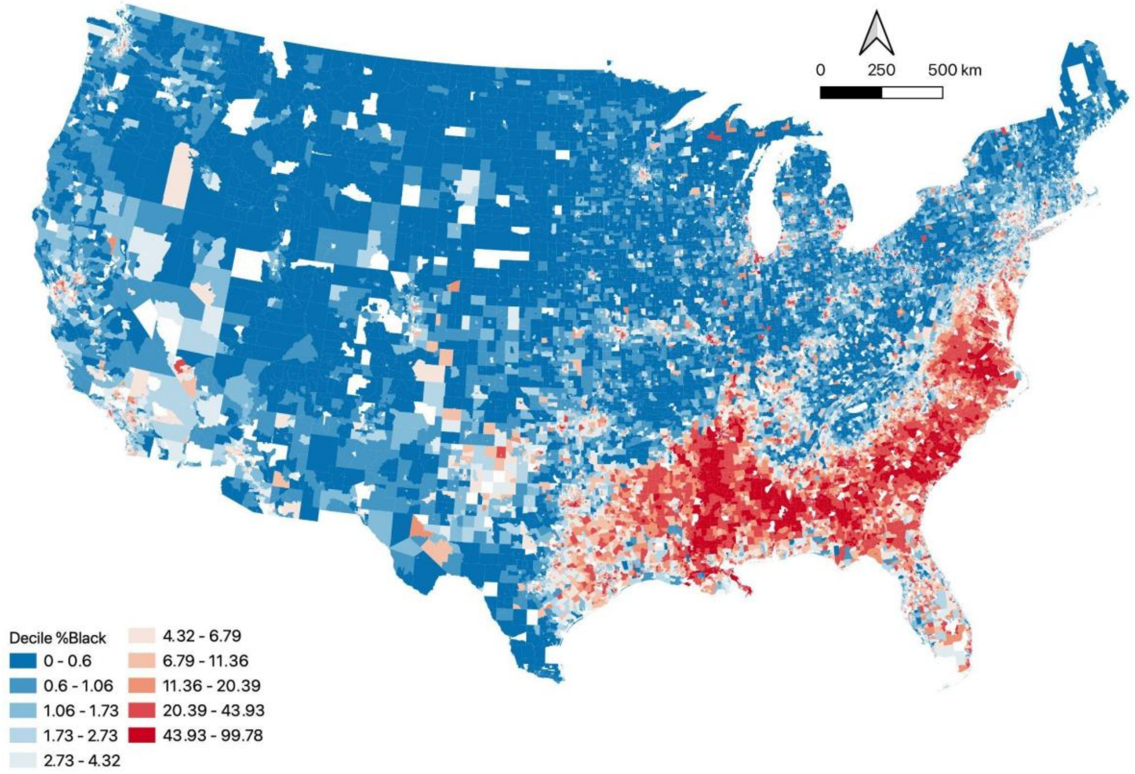

© 2022 Boing AF et al. *JAMA Network Open.*

eTable 1. Variance in Probability of Dying by Age Groups and Variance Change From Null Model When Adjusted by Census Tract Concentration $PM_{2.5}$ and Socioeconomic and Demographic Variables*

	State			County			Census tract		
Age	VE	SE	% VE explained	VE	SE	% VE explained	VE	SE	% VE explained
Under 1	0.349	0.091	76.072	0.527	0.059	59.202	38.107	0.211	5.022
1-4	0.025	0.007	74.665	0.025	0.005	83.425	2.947	0.016	2.053
5-14	0.019	0.005	81.341	0.029	0.006	78.153	3.636	0.020	2.739
15-24	0.449	0.115	66.075	1.166	0.085	44.714	25.785	0.143	5.144
25-34	1.481	0.350	67.133	3.598	0.241	52.213	57.133	0.319	9.479
35-44	3.601	0.806	78.872	7.596	0.472	59.991	102.915	0.574	24.018
45-54	11.066	2.503	86.417	22.628	1.332	65.190	292.849	1.633	40.330
55-64	24.522	5.667	90.503	42.296	2.624	73.006	777.204	4.316	46.488
65-74	81.128	18.350	84.124	94.870	6.579	72.712	2479.139	13.740	34.503
75-84	270.094	59.897	64.579	213.981	17.823	52.112	9620.550	53.169	8.384

*: Adjusted by census tract % of black people, % of people aged 25 or older with a college degree, median household income, % of residents below the federal poverty line, and population density.

© 2022 Boing AF et al. *JAMA Network Open.*

eTable 2. Variance in Probability of Dying by Age Groups and Variance Change From Null Model When Adjusted by Census Tract Socioeconomic and Demographic Variables*

	State			County			Census tract		
Age	VE	SE	% VE explained	VE	SE	% VE explained	VE	SE	% VE explained
Under 1	0.434	0.106	70.255	0.527	0.058	59.208	38.041	0.210	5.188
1-4	0.025	0.006	74.178	0.024	0.005	83.726	2.947	0.016	2.059
5-14	0.021	0.006	78.970	0.028	0.005	79.224	3.668	0.020	1.863
15-24	0.434	0.111	67.190	1.204	0.086	42.904	25.741	0.143	5.306
25-34	1.406	0.327	68.805	3.687	0.242	51.031	57.014	0.317	9.668
35-44	3.546	0.783	79.195	7.561	0.466	60.171	102.844	0.572	24.071
45-54	12.680	2.785	84.435	21.320	1.273	67.202	293.528	1.631	40.191
55-64	32.246	7.202	87.512	40.223	2.534	74.329	778.906	4.311	46.371
65-74	101.678	22.144	80.103	92.885	6.470	73.283	2479.480	13.698	34.494
75-84	322.865	70.232	57.659	216.906	17.854	51.457	9610.092	52.949	8.484

*: Adjusted by census tract % of black people, % of people aged 25 or older with a college degree, median household income, % of residents below the federal poverty line, and population density.

© 2022 Boing AF et al. *JAMA Network Open.*

eTable 3. Variance in Probability of Dying by Age Groups, and Percentage of Variance Change From the Null Model When Adjusted by Census Tract Concentration of $PM_{2.5}$

	State			County			Census tract		
Age	VE	SE	% VE explained	VE	SE	% VE explained	VE	SE	% VE explained
Under 1	0.979	0.226	32.916	1.425	0.104	-10.354	39.965	0.222	0.391
1-4	0.107	0.025	-9.036	0.154	0.012	-2.283	3.007	0.017	0.060
5-14	0.106	0.025	-5.502	0.137	0.013	-1.960	3.705	0.021	0.887
15-24	1.478	0.334	-11.745	2.059	0.127	2.369	27.194	0.152	-0.037
25-34	4.965	1.079	-10.171	7.522	0.383	0.102	63.109	0.352	0.010
35-44	14.545	3.144	14.649	25.311	1.081	-33.320	131.772	0.736	2.714
45-54	63.757	13.688	21.736	105.022	4.165	-61.558	459.869	2.568	6.298
55-64	171.814	36.847	33.461	249.670	10.126	-59.340	1352.591	7.539	6.872
65-74	291.599	64.045	42.939	487.673	21.365	-40.272	3602.788	20.053	4.817
75-84	377.245	83.099	50.527	475.715	28.924	-6.463	10395.230	57.566	1.007

Red: variance increased

© 2022 Boing AF et al. *JAMA Network Open.*

eTable 4. Crude and Adjusted Multilevel Regression Coefficient of $PM_{2.5}$ and Socioeconomic and Demographic Variables*

	Crude β coefficient (CI‡95%)	Adjusted** β coefficient (CI95%)
Population density		
Quintile 1 (lowest)	1.00	1.00
Quintile 2	0.58 (0.56;0.60)	0.56 (0.54;0.58)
Quintile 3	1.35 (1.32;1.37)	1.29 (1.27;1.31)
Quintile 4	1.90 (1.88;1.92)	1.80 (1.77;1.82)
Quintile 5 (highest)	2.26 (2.23;2.28)	2.08 (2.05;2.10)
Proportion of black residents		
Quintile 1 (lowest)	1.00	1.00
Quintile 2	0.41 (0.40;0.44)	0.10 (0.08;0.11)
Quintile 3	0.70 (0.67;0.72)	0.15 (0.13;0.17)
Quintile 4	0.84 (0.82;0.87)	0.11 (0.09;0.14)
Quintile 5 (highest)	0.97 (0.94;1.00)	0.08 (0.05;0.10)
Proportion of residents below the federal poverty line		
Quintile 1 (lowest)	1.00	1.00
Quintile 2	0.20 (0.18;0.22)	0.04 (0.03;0.06)
Quintile 3	0.35 (0.33;0.37)	0.05 (0.03;0.07)
Quintile 4	0.53 (0.51;0.55)	0.07 (0.05;0.09)
Quintile 5 (highest)	0.79 (0.76;0.81)	0.12 (0.09;0.14)
Median household income		
Quintile 1 (lowest)	1.00	
Quintile 2	-0.27 (-0.29;-0.25)	-0.04 (-0.06;-0.02)
Quintile 3	-0.46 (-0.49;-0.44)	-0.05 (-0.07;-0.03)
Quintile 4	-0.65 (-0.67;-0.63)	-0.08 (-0.10;-0.05)
Quintile 5 (highest)	-0.92 (-0.94;-0.89)	-0.15 (-0.18;-0.12)
Proportion of people aged 25 or older who have college degree		
Quintile 1 (lowest)	1.00	1.00
Quintile 2	-0.21 (-0.23;-0.18)	-0.08 (-0.10;-0.06)
Quintile 3	-0.28 (-0.30;-0.26)	-0.12 (-0.14;-0.10)
Quintile 4	-0.37 (-0.40;-0.35)	-0.16 (-0.18;-0.14)
Quintile 5 (highest)	-0.50 (-0.52;-0.47)	-0.18 (-0.20;-0.15)

*: The outcome particulate matter ($PM_{2.5}$) concentration was analyzed as a continuous variable.
**: Adjusted by all variables
‡: Confidence Interval

© 2022 Boing AF et al. *JAMA Network Open.*

eTable 5. Crude and Adjusted* Multilevel Regression Coefficient of Probability of Dying (×1000) According to Census Tract $PM_{2.5}$ as a Continuous Variable

	β coefficient* ($CI_{95\%}$)	
	Crude	Adjusted
Under 1	0.361 (0.319;0.403)	0.105 (0.059;0.151)
1-4	0.006 (-0.006; 0.018)	0.009 (-0.003;0.021)
5-14	-0.005 (-0.018;0.007)	0.014 (0.001;0.027)
15-24	0.014 (-0.024;0.052)	0.016 (-0.027;0.060)
25-34	0.068 (0.008;0.129)	0.028 (-0.039;0.096)
35-44	1.559 (1.468;1.651)	0.087 (-0.006;0.180)
45-54	5.067 (4.894;5.240)	0.631 (0.474;0.788)
55-64	9.374 (9.082;9.666)	1.366 (1.119;1.613)
65-74	12.864 (12.402;13.327)	2.073 (1.645;2.502)
75-84	10.122 (9.420;10.824)	1.603 (0.803;2.403)

*: Adjusted by census tract % of black people, % of people aged 25 or older with a college degree, median household income, % of residents below the federal poverty line, and population density.

© 2022 Boing AF et al. *JAMA Network Open.*

eTable 6. Crude and Adjusted* Multilevel Regression Coefficient of Probability of Dying According to Census Tract Concentration of $PM_{2.5}$ Deciles

Age	β coefficient* ($CI_{95\%}$)								
	Decile 2	Decile 3	Decile 4	Decile 5	Decile 6	Decile 7	Decile 8	Decile 9	Decile 10
**Under 1**									
Crude	0.187	0.295	0.774	0.984	0.994	1.129	1.163	2.454	3.124
Adjusted	0.108	0.015	0.290	0.234	0.098	0.213	0.401	0.777	1.014
**1-4**									
Crude	-0.031	-0.061	-0.062	-0.036	-0.072	-0.091	-0.134	0.043	0.159
Adjusted	0.048	0.042	0.041	0.053	0.031	0.058	-0.006	0.103	0.119
**5-14**									
Crude	-0.116	-0.128	-0.109	-0.137	-0.146	-0.201	-0.162	-0.091	0.051
Adjusted	-0.007	0.011	0.038	-0.000	0.007	0.011	0.040	0.058	0.122
**15-24**									
Crude	-0.760	-0.815	-0.922	-0.763	-0.820	-0.742	-0.801	-0.116	0.716
Adjusted	-0.441	-0.424	-0.493	-0.401	-0.435	-0.180	-0.322	0.135	0.672
**25-34**									
Crude	-0.637	-0.532	-0.778	-0.929	-0.628	-0.964	-0.742	0.331	1.702
Adjusted	-0.210	-0.018	-0.169	-0.425	-0.099	-0.111	-0.118	0.499	1.304
**35-44**									
Crude	0.818	2.120	3.265	4.283	5.281	5.556	7.671	10.379	13.468
Adjusted	-0.196	-0.173	-0.235	-0.316	-0.048	-0.065	0.471	1.067	2.218
**45-54**									
Crude	4.739	9.680	13.713	16.973	20.020	21.420	26.788	34.414	41.865
Adjusted	0.825	1.621	1.907	1.823	2.641	2.644	3.686	5.637	7.914
**55-64**									
Crude	10.149	19.223	26.645	31.881	37.175	39.974	49.506	63.133	76.099
Adjusted	2.824	4.317	4.832	4.111	5.382	5.778	7.632	10.817	13.998
**65-74**									
Crude	16.274	30.206	38.765	45.571	52.934	54.386	67.060	87.243	102.685
Adjusted	5.229	8.480	7.828	7.193	9.149	7.694	10.164	16.256	18.566
**75-84**									
Crude	22.078	33.159	38.533	43.572	48.188	47.254	60.619	71.363	75.633
Adjusted	10.372	12.428	10.756	10.572	10.415	7.478	13.226	13.826	9.777

*: Adjusted by census tract % of black people, % of people aged 25 or older with a college degree, median household income, % of residents below the federal poverty line, and population density.
Blue shades: $p<0.05$; orange shades: $p>0.05$

© 2022 Boing AF et al. *JAMA Network Open.*

eTable 7. Multilevel Regression Coefficient of Probability of Dying According to Census Tract Concentration of $PM_{2.5}$ Deciles and Median Household Income Quintiles

Age group	β coefficient* ($CI_{95\%}$)								
	Decile 2	Decile 3	Decile 4	Decile 5	Decile 6	Decile 7	Decile 8	Decile 9	Decile 10
**45-54 years**									
Quintile 1	3.885	6.679	6.680	9.404	11.192	15.094	16.703	18.009	21.926
Quintile 2	3.536	6.947	7.778	8.021	9.399	8.804	9.646	11.159	11.029
Quintile 3	2.559	4.093	4.765	5.057	5.888	5.092	5.362	6.702	6.246
Quintile 4	1.360	2.383	3.105	3.438	3.213	2.494	3.429	4.190	4.144
Quintile 5	0.336	0.818	1.576	2.162	2.751	2.556	3.099	4.292	3.654
**55-64 years**									
Quintile 1	10.402	16.509	19.160	22.557	27.112	32.379	39.167	41.040	45.167
Quintile 2	9.765	15.783	17.182	20.131	20.571	21.456	22.795	26.553	25.728
Quintile 3	6.933	9.984	13.948	14.297	15.913	15.136	14.735	17.126	18.537
Quintile 4	5.732	8.362	10.400	10.054	9.937	9.487	9.533	11.477	12.450
Quintile 5	1.472	2.776	3.810	4.984	6.679	6.059	6.963	8.042	8.858
**65-74 years**									
Quintile 1	13.313	22.425	28.471	33.744	38.287	38.387	51.334	52.425	52.773
Quintile 2	18.303	30.813	30.888	34.919	37.471	38.478	41.417	45.004	43.927
Quintile 3	13.149	18.278	24.274	26.870	28.597	27.537	25.074	30.504	35.008
Quintile 4	9.987	17.607	22.438	21.552	19.703	20.341	20.396	25.623	26.224
Quintile 5	6.904	9.124	11.518	11.661	15.189	13.760	13.217	16.117	15.603
**75-84 years**									
Quintile 1	24.227	31.260	29.418	40.596	33.723	40.323	47.517	50.308	44.114
Quintile 2	25.332	37.464	39.950	44.600	47.414	44.261	50.178	56.177	50.483
Quintile 3	25.639	31.276	44.424	41.814	45.779	42.421	45.896	51.601	49.973
Quintile 4	22.515	31.855	35.733	39.411	35.196	37.671	37.496	43.881	41.835
Quintile 5	5.969	11.774	4.568	10.123	19.953	5.909	12.729	12.295	6.607

Blue shades: $p<0.05$; orange shades: $p>0.05$

© 2022 Boing AF et al. *JAMA Network Open.*

eTable 8. Multilevel Regression Coefficient of Probability of Dying According to Census Tract Concentration of $PM_{2.5}$ Deciles and Proportion of Black Residents

Age group	β coefficient* ($CI_{95\%}$)								
	Decile 2	Decile 3	Decile 4	Decile 5	Decile 6	Decile 7	Decile 8	Decile 9	Decile 10
**45-54 years**									
Quintile 1	1.019	3.615	4.077	2.723	2.724	2.232	2.756	3.994	8.081
Quintile 2	4.034	6.553	6.827	6.882	7.295	8.535	9.697	10.901	13.884
Quintile 3	5.633	8.273	10.077	10.974	11.047	13.269	13.461	16.338	18.322
Quintile 4	5.277	9.571	10.952	13.016	14.897	17.349	19.903	24.187	28.963
Quintile 5	-4.195	0.361	4.126	6.967	10.798	15.947	19.962	29.542	39.420
**55-64 years**									
Quintile 1	4.050	8.134	9.612	7.702	7.761	7.654	10.551	12.135	21.977
Quintile 2	7.995	11.421	12.788	12.452	13.952	15.389	16.870	19.646	28.932
Quintile 3	8.957	12.730	18.307	17.511	18.884	22.596	22.033	28.590	31.348
Quintile 4	11.184	19.009	23.329	24.931	26.399	31.273	35.677	44.334	52.324
Quintile 5	-3.897	9.021	12.466	19.444	26.097	34.076	42.030	55.904	71.733
**65-74 years**									
Quintile 1	7.672	14.771	18.216	14.087	11.708	17.449	18.060	18.486	32.661
Quintile 2	10.895	16.622	16.989	15.133	16.135	18.518	21.641	27.583	38.160
Quintile 3	11.900	14.081	23.163	22.546	22.827	27.237	26.633	34.367	42.990
Quintile 4	9.508	21.950	26.453	27.449	30.367	33.361	38.932	48.756	58.043
Quintile 5	-2.070	12.413	15.013	25.073	32.321	37.351	46.841	64.072	77.044
**75-84 years**									
Quintile 1	16.403	26.853	25.765	18.579	18.930	25.475	24.092	25.281	40.215
Quintile 2	10.977	11.537	7.703	11.733	16.850	9.356	15.992	20.785	27.338
Quintile 3	7.379	10.596	17.123	16.621	12.724	9.270	15.277	21.899	20.471
Quintile 4	14.454	13.706	15.445	17.956	16.324	16.928	17.564	25.543	29.457
Quintile 5	-23.488	-10.723	-14.597	-8.772	-6.307	-4.140	5.373	17.657	16.208

Blue shades: $p<0.05$; orange shades: $p>0.05$

© 2022 Boing AF et al. *JAMA Network Open.*

eTable 9. Multilevel Regression Coefficient of Probability of Dying According to Census Tract Concentration of $PM_{2.5}$ Deciles and Share of Residents Below the Federal Poverty Line Quintiles

Age group	β coefficient* ($CI_{95\%}$)								
	Decile 2	Decile 3	Decile 4	Decile 5	Decile 6	Decile 7	Decile 8	Decile 9	Decile 10
**45-54 years**									
Quintile 1	1.031	2.558	3.363	3.789	4.796	4.858	6.027	7.431	8.366
Quintile 2	0.963	3.080	4.216	4.306	4.266	4.849	6.765	7.933	9.214
Quintile 3	2.160	3.722	4.535	5.151	5.143	4.787	5.637	7.655	8.717
Quintile 4	6.503	8.005	8.132	9.406	9.840	9.206	13.674	13.030	6.503
Quintile 5	2.364	5.890	6.174	8.838	11.628	15.017	16.993	18.977	22.702
**55-64 years**									
Quintile 1	2.999	5.188	7.999	8.929	9.971	9.783	11.336	14.761	16.655
Quintile 2	3.988	7.110	9.825	9.831	11.252	11.573	12.743	16.295	20.710
Quintile 3	6.945	11.488	12.706	13.955	14.159	14.906	16.772	18.701	21.507
Quintile 4	9.214	14.938	18.135	18.793	19.947	22.025	24.038	29.413	28.859
Quintile 5	7.719	15.779	18.831	23.473	28.494	34.059	38.641	42.901	46.674
**65-74 years**									
Quintile 1	9.060	12.994	18.376	18.603	21.086	21.264	22.675	26.333	30.924
Quintile 2	7.956	14.481	20.159	20.443	20.688	20.452	26.967	27.746	36.248
Quintile 3	10.950	19.314	21.780	22.971	25.019	26.508	24.808	32.633	36.396
Quintile 4	15.622	26.529	32.326	33.871	34.803	35.242	36.444	49.584	44.294
Quintile 5	13.731	24.947	26.960	32.919	37.724	40.213	49.434	52.120	52.903
**75-84 years**									
Quintile 1	17.926	23.028	22.839	23.277	31.257	24.811	27.719	31.435	30.563
Quintile 2	18.620	27.083	31.122	33.405	34.783	33.193	32.991	41.678	41.791
Quintile 3	20.738	29.847	32.468	39.336	37.775	38.091	42.585	42.985	47.350
Quintile 4	25.946	33.313	43.986	44.681	50.189	41.109	47.537	60.912	55.108
Quintile 5	18.986	32.244	26.515	33.509	28.431	33.673	43.827	44.889	34.230

Blue shades: $p<0.05$; orange shades: $p>0.05$

© 2022 Boing AF et al. *JAMA Network Open.*

eTable 10. Multilevel Regression Coefficient of Probability of Dying According to Census Tract Concentration of $PM_{2.5}$ Deciles and Population Density Quintiles

Age group	β coefficient* ($CI_{95\%}$)								
	Decile 2	Decile 3	Decile 4	Decile 5	Decile 6	Decile 7	Decile 8	Decile 9	Decile 10
**45-54 years**									
Quintile 1	-1.160	0.428	0.149	-1.071	-1.515	-2.604	-1.620	-4.961	0.682
Quintile 2	1.171	3.325	4.440	5.716	8.692	11.541	13.411	15.934	20.888
Quintile 3	4.771	7.895	9.897	11.354	15.045	19.528	24.539	30.915	38.202
Quintile 4	8.537	12.440	14.520	16.806	20.406	22.559	24.572	31.879	37.315
Quintile 5	5.340	12.719	15.142	17.355	19.123	20.647	19.756	24.016	30.973
**55-64 years**									
Quintile 1	1.064	5.729	6.320	4.234	4.098	3.832	6.063	2.910	7.188
Quintile 2	1.461	4.152	7.445	8.550	14.152	17.599	23.501	26.801	38.165
Quintile 3	8.720	13.636	18.548	21.075	28.921	33.998	43.766	54.563	68.447
Quintile 4	16.867	23.439	27.355	30.975	36.061	39.616	44.327	54.998	63.019
Quintile 5	14.139	28.962	31.219	34.001	38.345	44.512	41.754	52.787	59.667
**65-74 years**									
Quintile 1	0.982	9.720	11.566	9.238	11.674	12.583	13.751	10.598	3.205
Quintile 2	2.847	7.913	13.328	17.658	22.270	29.469	35.377	38.670	52.288
Quintile 3	9.464	15.033	20.645	24.546	33.626	35.104	49.765	62.217	81.977
Quintile 4	24.878	33.145	37.107	38.968	47.841	51.920	56.773	73.785	82.617
Quintile 5	18.702	43.431	48.627	45.689	50.698	56.902	52.369	67.065	72.036
**75-84 years**									
Quintile 1	11.067	19.827	26.887	23.781	28.417	24.612	21.062	26.581	12.602
Quintile 2	4.886	12.812	12.411	16.600	17.430	21.374	20.962	19.280	34.647
Quintile 3	12.120	20.319	15.088	14.724	25.319	28.191	35.418	44.601	53.256
Quintile 4	15.115	16.584	18.609	25.812	28.643	26.607	31.309	45.284	42.650
Quintile 5	9.617	25.629	21.580	22.315	21.965	18.954	33.169	42.977	41.530

Blue shades: $p<0.05$; orange shades: $p>0.05$

© 2022 Boing AF et al. *JAMA Network Open.*

eTable 11. Multilevel Regression Coefficient of Probability of Dying According to Census Tract Concentration of $PM_{2.5}$ Deciles and Quintiles of the Proportion of People Aged 25 Years or Older With a College Degree

Age group	β coefficient* ($CI_{95\%}$)								
	Decile 2	Decile 3	Decile 4	Decile 5	Decile 6	Decile 7	Decile 8	Decile 9	Decile 10
**45-54 years**									
Quintile 1	1.439	5.006	5.864	7.869	12.766	17.144	19.286	25.473	26.750
Quintile 2	2.818	6.474	7.973	10.523	12.746	16.934	17.868	21.490	24.416
Quintile 3	3.287	6.312	8.374	9.816	11.198	11.899	15.448	17.702	20.746
Quintile 4	2.689	5.612	7.466	9.469	11.133	12.617	14.651	16.720	19.810
Quintile 5	2.717	4.457	6.411	7.021	8.360	9.522	11.492	13.682	15.180
**55-64 years**									
Quintile 1	7.035	14.793	19.227	22.108	33.257	39.816	44.290	55.513	54.128
Quintile 2	8.116	14.882	19.795	22.478	26.518	32.131	37.030	44.174	49.756
Quintile 3	7.650	13.382	17.908	21.713	23.858	27.043	32.086	34.875	39.415
Quintile 4	7.312	11.937	14.068	18.296	19.789	23.568	26.366	31.350	36.520
Quintile 5	5.433	8.375	11.696	12.960	15.372	16.954	20.204	22.713	27.311
**65-74 years**									
Quintile 1	10.181	21.461	24.874	28.203	41.889	45.598	57.834	69.417	67.901
Quintile 2	14.399	26.639	30.499	38.302	44.372	49.099	52.850	65.393	63.820
Quintile 3	14.093	21.812	33.252	37.317	35.635	40.537	46.269	51.408	61.996
Quintile 4	13.076	22.593	27.492	31.932	35.354	40.310	43.179	51.321	55.668
Quintile 5	9.918	14.449	19.226	19.625	23.932	25.259	29.345	31.752	37.376
**75-84 years**									
Quintile 1	17.858	29.838	29.946	42.125	41.931	45.443	54.968	65.212	65.564
Quintile 2	23.106	34.442	41.000	45.289	49.125	59.570	55.807	72.484	64.294
Quintile 3	23.016	33.682	42.243	40.539	42.904	44.770	56.364	59.687	54.581
Quintile 4	21.495	24.727	30.311	35.950	38.564	35.315	42.871	45.965	35.984
Quintile 5	19.526	26.280	20.457	27.235	35.970	27.972	34.976	35.031	37.690

Blue shades: $p<0.05$; orange shades: $p>0.05$

© 2022 Boing AF et al. *JAMA Network Open.*
